# CHAS infers cell type-specific signatures in bulk brain histone acetylation studies of neurological and psychiatric disorders

**DOI:** 10.1016/j.crmeth.2025.101032

**Published:** 2025-04-28

**Authors:** Kitty B. Murphy, Yuqian Ye, Maria Tsalenchuk, Alexi Nott, Sarah J. Marzi

**Affiliations:** 1 UK Dementia Research Institute at King’s College London, London, UK; 2 Department of Basic and Clinical Neuroscience, Institute of Psychiatry, Psychology and Neuroscience, King’s College London, London, UK; 3 Department of Brain Sciences, Imperial College London, London, UK; 4 UK Dementia Research Institute at Imperial College London, London, UK

**Keywords:** cell types, histone acetylation, epigenetics, neurodegeneration, brain disorders, deconvolution, brain

## Abstract

Epigenomic profiling of the brain has largely been done on bulk tissues, limiting our understanding of cell type-specific epigenetic changes in disease states. Here, we introduce cell type-specific histone acetylation score (CHAS), a computational tool for inferring cell type-specific signatures in bulk brain H3K27ac profiles. We applied CHAS to >300 H3K27ac chromatin immunoprecipitation sequencing samples from studies of Alzheimer’s disease, Parkinson’s disease, autism spectrum disorder, schizophrenia, and bipolar disorder in bulk postmortem brain tissue. In addition to recapitulating known disease-associated shifts in cellular proportions, we identified cell type-specific biological insights into brain-disorder-associated regulatory variation. In most cases, genetic risk and epigenetic dysregulation targeted different cell types, suggesting independent mechanisms. For instance, genetic risk of Alzheimer’s disease was exclusively enriched within microglia, while epigenetic dysregulation predominantly fell within oligodendrocyte-specific H3K27ac regions. In addition, reanalysis of the original datasets using CHAS enabled identification of biological pathways associated with each neurological and psychiatric disorder at cellular resolution.

## Highlights

• CHAS is a cell type deconvolution tool for bulk brain histone acetylation profiles
• Genetic risk and epigenetic dysregulation target different cell types
• CHAS is adaptable to other reference and bulk datasets
• CHAS is an open-source R package

## Motivation

Acetylation of histone H3 lysine 27 (H3K27ac) has emerged as an informative disease-associated epigenetic mark. However, cell type-specific contributions to epigenetic dysregulation in disease are unclear as studies have often used bulk brain tissue. Therefore, methods for the deconvolution of bulk H3K27ac profiles are critical. Here, we introduce cell type-specific histone acetylation score (CHAS), a computational tool for cell type deconvolution of bulk brain H3K27ac profiles. CHAS is implemented as an open-source R package.

## Graphical abstract

**Figure:**
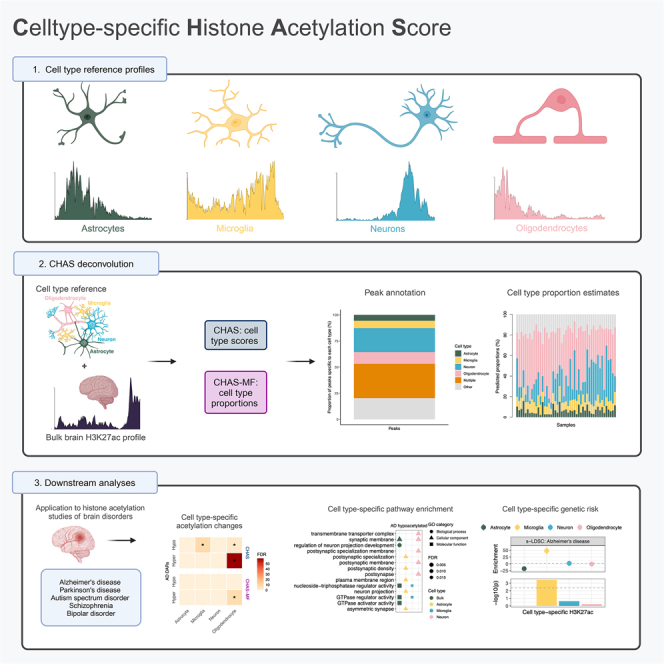

## Teaser

Murphy et al. develop CHAS, a computational tool that uncovers cell type-specific patterns in bulk tissue histone acetylation data. Applying CHAS to brain disorders reveals distinct cell type targets of genetic risk and epigenetic changes. The tool is adaptable to various tissues and available as an R package.

## Introduction

H3K27ac is a highly cell type-specific epigenetic modification that marks active enhancers and promoters.^1^ Brain disorder risk variants predominantly fall into non-coding and regulatory regions,^2^ such as those marked by H3K27ac.^3^ Integrating genome-wide profiles of H3K27ac from disease-relevant cell types can be useful for functional interpretation of these risk variants. This was demonstrated by efforts mapping regulatory elements to major cell types in the human cortex and investigating neurological and psychiatric disease-risk associations.^4^ Identifying cell type-specific H3K27ac signals in brain disorders can therefore be used to infer dysregulated signaling pathways at cell type resolution.

Studies on postmortem human brains have identified genome-wide dysregulation of histone acetylation associated with several brain disorders.^5^^,^^6^^,^^7^^,^^8^^,^^9^ However, interpretation of these studies is limited by the use of bulk tissue, which does not account for cellular heterogeneity in the brain. This can lead to biological findings being driven by differences in cellular abundance rather than disease-associated changes, and limits follow-up studies in the appropriate cell types. To control for cellular composition, studies have used approaches such as CETS,^10^ a tool for quantifying neuronal proportions from DNA methylation data, and by measuring the neuronal fraction using flow cytometry. However, these methods require DNA methylation profiles for samples from the same individuals and generally only estimate the proportion of neuronal cell types vs. non-neuronal cell types, rather than individual glial cell types.

Although H3K27ac profiling at the purified cell or nuclei population and single-cell level is gaining traction,^11^^,^^12^ only a few studies have been applied to the human brain,^13^ with most only characterizing NeuN^+^ and NeuN^−^ samples.^8^^,^^9^^,^^14^ This highlights an opportunity for the development of cell type deconvolution methods to better interpret bulk brain H3K27ac profiles. Currently, only one approach has been described for this purpose.^15^

Here, we present CHAS (cell type-specific histone acetylation score), a method for cell type deconvolution of bulk brain histone acetylation profiles. To ensure robustness of our model, we implemented two independent algorithms, herein referred to as CHAS and CHAS-MF, and compared their performance across multiple validation approaches and bulk brain histone acetylation studies. We applied both algorithms to five brain disorder H3K27ac datasets: Alzheimer’s disease (AD),^6^ Parkinson’s disease (PD),^8^ autism spectrum disorder (ASD),^5^ schizophrenia (SCZ), and bipolar disorder (BPD), to detect shifts in cellular composition and re-investigate differential histone acetylation while controlling for cell type composition. In addition, CHAS allowed identification of cell type-specific genetic risk and epigenetic dysregulation enrichments, which overall highlighted distinct cell types. CHAS is implemented as an open-source R package.

## Results

### The CHAS model

#### CHAS

Enhancers and H3K27ac domains are known to be cell type specific.^4^^,^^16^^,^^17^^,^^18^ CHAS leverages this to annotate peaks identified in bulk brain studies of H3K27ac to their cell type-specific signals in neurons, microglia, oligodendrocytes, and astrocytes.^4^ Although we focused on one brain cell reference dataset to perform validation, CHAS can be used with any bulk and reference datasets. CHAS achieves this by overlapping bulk brain H3K27ac peaks with each cell type-specific peak set. For a bulk peak to be defined as cell type-specific, two criteria must be met: (1) the bulk peak is annotated only to a single cell type and (2) the bulk peak overlaps a predefined percentage of that cell type’s peak. This step outputs the bulk peaks annotated to each single cell type, “multiple” cell types (the peak is annotated to more than one cell type), and “other” (the bulk peak is not annotated to any of the reference cell types).

Bulk tissue analysis can be challenging due to differences in cell type proportion in response to disease, or from discrepancies in brain region sampling. To overcome this, using each set of cell type-specific H3K27ac peaks, CHAS generates cell type-specific scores. By averaging the normalized signal intensity of a sample across all peaks specific to a given cell type, CHAS derives a proxy of the proportion of that cell type in the bulk sample.

Given the application of CHAS to deconvolute and control for cellular heterogeneity in histone acetylation studies of brain disorders, we must work under the assumption that disease-related differences in histone acetylation are limited to only a subset of cell type-specific peaks, and that cell type-specific epigenetic variation far outweighs variation associated with disease status.^14^^,^^19^ We can therefore use cell type-specific chromatin immunoprecipitation sequencing (ChIP-seq) H3K27ac signal intensities as a proxy for cell type proportion in bulk tissue data.

CHAS requires three inputs:

(1) bulk tissue H3K27ac peaks.
(2) cell-sorted H3K27ac reference peaks.
(3) counts matrix for the bulk H3K27ac peaks.

**Figure 1:**
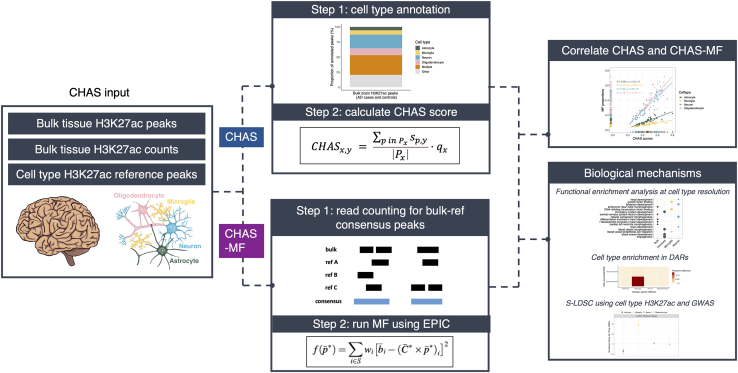
CHAS workflow CHAS is split into two main computational workflows: CHAS and CHAS-MF. The CHAS-derived cell type scores and MF-derived cell type proportions can be correlated, and both can be used as covariates in downstream analyses. *CHAS*x,y: cell type-specific score for cell type x, sample y; P x: set of cell type-specific peaks for cell type x; S p,y: standardized peak signal intensity for peak p, sample y. q x: normalization factor for cell type x. A constraint is applied to S p,y whereby for each peak p, the maximum peak signal intensity for any sample equals 1.

CHAS then performs two main analytical tasks (Figure 1):

(1) Identification of cell type-specific peaks in bulk tissue H3K27ac profiles using cell-sorted H3K27ac data.
(2) Generation of cell type-specific scores on the basis of genome-wide average ChIP-seq signal intensities.

#### CHAS-MF

We implemented a second algorithm in CHAS, CHAS-MF, to estimate cell type proportions using non-negative matrix factorization. Based on the EPIC R package,^20^ CHAS-MF models bulk counts as the sum of cell type-specific counts weighted by their respective proportions. It first identifies consensus peaks by merging bulk and reference peaks, then generates read counts for these peaks using either (1) bam files, if available, or (2) bulk and reference counts as proxies for consensus peak counts.

As EPIC was originally designed for transcriptomic data, we introduced three modifications to improve its accuracy for deconvoluting H3K27ac profiles: (1) bulk and reference H3K27ac counts are normalized based on peak length and library size, (2) if there are multiple reference samples for a given cell type, the median normalized counts per million (CPM) are used for deconvolution, and (3) weighting of peaks to account for read count and signal variability. Higher weights are assigned to peaks with strong, cell type-specific signals, while those with lower counts or higher variability receive lower weights. Instead of applying MF to all consensus peaks, CHAS-MF selects signature peaks, defined as those with high read counts in a single cell type and low counts in others.

CHAS-MF requires three inputs:

(1) bulk tissue H3K27ac peaks.
(2) cell-sorted H3K27ac reference peaks.
(3) either the bam files for bulk and reference samples or the counts matrices for the bulk and reference H3K27ac peaks.

CHAS-MF then performs three main tasks (Figure 1):

(1) Identification of consensus peaks by merging bulk and reference H3K27ac peaks.
(2) Generation of read counts for consensus peaks in bulk and reference H3K27ac samples.
(3) Prediction of cell type proportions using MF on the basis of normalized genome-wide ChIP-seq signal intensities.

### Validation of CHAS

**Figure 2:**
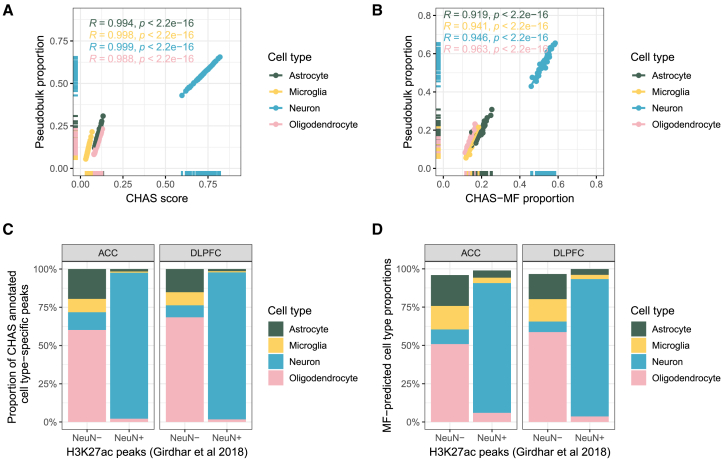
CHAS cell type scores and proportions correlate with true cell type proportions (A) Scatterplot of CHAS-derived cell type scores vs. true proportions in pseudobulk samples, with Spearman’s correlation and regression lines for each cell type. (B) Scatterplot of CHAS-MF proportions vs. true proportions in pseudobulk samples, with Spearman’s correlation and regression lines for each cell type. (C) Proportion of CHAS-annotated cell type-specific peaks in NeuN^−^ and NeuN^+^ H3K27ac profiles. (D) CHAS-MF cell type proportions in NeuN^−^ and NeuN^+^ H3K27ac profiles.

To validate CHAS’s accuracy in predicting cell type composition, we simulated pseudobulk H3K27ac profiles using sorted H3K27ac data. Each sample contained 30 million randomly sampled reads from astrocytes, microglia, neurons, and oligodendrocytes, with compositions based on cortical proportions from AD 17 and 33 non-AD individuals.^21^ After peak calling and read count generation, CHAS showed near-perfect correlation with true cell type proportions (Spearman’s rank correlation coefficient, R ≥ 0.99, *p* < 2.2 × 10^−16^ for CHAS and R ≥ 0.92, *p* < 2.2 × 10^−16^ for CHAS-MF across all cell types, Figures 2A and 2B). Robustness was confirmed across varying read depths (10M, 20M) and sample sizes (10, 25), with consistently strong correlations (Spearman’s rank correlation coefficient, R ≥ 0.99 for each analysis; Figures S1A–S1D). Further validation was performed using CHAS and CHAS-MF to deconvolute NeuN^+^ and NeuN^−^ H3K27ac data from two different brain regions of 15 healthy individuals.^14^ In the anterior cingulate cortex (ACC), mean neuronal proportions were significantly higher in NeuN^+^ vs. NeuN^−^ samples (CHAS, Welch’s t test, mean difference = 0.86, *p* = 4.35 × 10^−14^; CHAS-MF, paired t test, mean difference = 0.85, *p* < 2.2 × 10^−16^; Figure 2C). Similar results were observed in dorsolateral prefrontal cortex (DLPFC) samples (CHAS, paired t test, mean difference = 0.89, *p* < 2.2 × 10^−16^; CHAS-MF, paired t test, mean difference = 0.90, *p* < 2.2 × 10^−16^; Figure 2D).

As an additional performance metric, we assessed to what extent CHAS can handle new, rare, and missing cell types. Microglia make up to 15% of the cellular composition depending on brain region^22^ and were the rarest cell type included in our study. In our pseudobulk samples, the microglial proportion ranged from 5% to 21% and CHAS showed strong performance across these proportions (Figures 2A, 2B, and S1). To test whether CHAS could handle new cell types, we ran CHAS using the same 49 pseudobulk samples described previously excluding microglia from the cell type reference data. This did not affect CHAS’s ability to calculate cell type scores and proportions that were strongly correlated with the true proportions (Figures S2A and S2B). Finally, we generated 46 pseudobulk samples made up of 30 million randomly sampled reads from astrocytes, neurons, and oligodendrocytes. This enabled us to evaluate the performance of CHAS when a cell type is missing from the bulk sample but is present in the cell type reference data. The correlation between the pseudbulk proportions and CHAS-derived scores as well as proportions remained significant (Figures S2C and S2D). Furthermore, we wanted to evaluate whether CHAS could resolve cell subtypes. To test this, we used H3K27ac profiles for glutamatergic and GABAergic neurons,^23^ with NeuN^+^ data. CHAS-annotation revealed ∼70% annotation to glutamatergic and ∼30% annotation to GABAergic signatures, as might be expected from a cortical sample^24^ (Figure S2E). To test whether these signatures were subtype-specific and not reflective of a general neuronal signature, we included neurons, as well as the other reference cell types in CHAS. CHAS was still able to detect cell subtype-specific peaks within the NeuN^+^ profiles (Figure S2F). Finally, we explored the limit of how many cell type-specific peaks in the reference data were sufficient to characterize rarer cell types such as microglia. Using the 49 pseudobulk samples previously described, we downsampled the cell type reference peaks by 25%, 50%, 75%, and 90%. The largest effect was seen with neurons, the most abundant cell type in our reference data, with performance dropping with the first downsampling of 25%. For microglia, our rarest cell type, performance started to drop marginally after 50%. However, even at 90% downsampling we were able to capture microglia-specific peaks in the pseudobulk samples (Figure S2G).

### Deconvolution of bulk brain H3K27ac in AD highlights oligodendrocyte-specific epigenetic dysregulation

Aberrations in H3K27ac associated with AD have been reported in the human brain^6^^,^^7^; however, the contribution of individual cell types in epigenetic dysregulation associated with the disease is only beginning to be dissected.^13^ To address this, we used CHAS to deconvolute H3K27ac profiles from the entorhinal cortex of AD 24 cases and 23 controls.^6^

**Figure 3:**
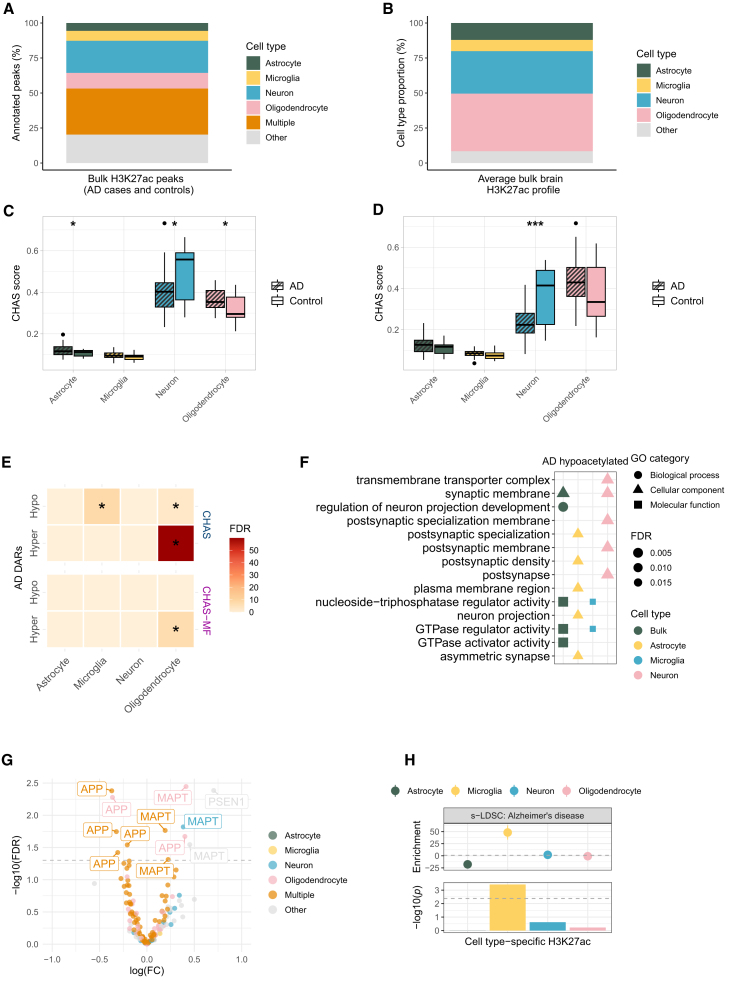
Cell type deconvolution of H3K27ac profiles from the AD brain highlights a role for oligodendrocytes and microglia (A) Stacked barplot of bulk brain H3K27ac peak annotations (AD cases and controls) to astrocytes, microglia, neurons, oligodendrocytes, multiple cell types, or other using CHAS. (B) Stacked barplot of average cell type proportions in bulk brain H3K27ac profiles (AD cases and controls) estimated by CHAS-MF. (C) Boxplot comparing CHAS cell type scores between AD cases and controls (Welch’s t test, ∗ *p* < 0.05, ∗∗ *p* < 0.01, ∗∗∗ *p* < 0.001, ∗∗∗∗ *p* < 0.0001). (D) Boxplot comparing CHAS-MF cell type proportions between AD cases and controls (Welch’s t test, ∗ *p* < 0.05, ∗∗ *p* < 0.01, ∗∗∗ *p* < 0.001, ∗∗∗∗ *p* < 0.0001). (E) Heatmap of cell type enrichments among AD-associated DARs, controlling for CHAS scores and CHAS-MF proportions (hypergeometric test, ∗ FDR < 0.05). (F) Functional enrichment analysis of AD-associated hypoacetylated regions by cell type, controlling for CHAS scores. Top 10 enriched pathways shown. (G) Volcano plot of H3K27ac peaks annotated to AD genes (APP, PSEN1, PSEN2, MAPT), highlighting DARs. (H) s-LDSC results using AD GWAS^25^ and CHAS-annotated cell type-specific H3K27ac peaks. Top: LDSC enrichment values. Bottom: −log10(*p*) of coefficient *Z* scores, with Bonferroni significance threshold (dashed line). See also Figure S5 and Table S1.

In this study, we re-analyzed the original data using CHAS. Of 183,353 peaks, 80% (*n* = 146,144) were annotatable to one or more cell types, with 47% (*n* = 85,824) specific to a single cell type (Figure 3A). Using CHAS-MF, we then estimated cell type proportions in each bulk sample, and found that the four cell types constituted on average 92% of the samples (Figure 3B). In the original study, neuronal proportion estimates for samples from the same individuals had been derived based on matched bulk brain DNA methylation data using CETS,^6^^,^^10^ a tool for estimating neuronal proportion. CHAS-derived neuronal scores and CETS-derived neuronal proportion estimates correlated across the 47 samples (Spearman’s rank correlation coefficient, R = 0.409, *p* = 0.00429, Figure S1E).

We next used CHAS to evaluate shifts in cellular composition in the bulk brain data, testing whether these replicate known disease-associated changes. To this end, we compared CHAS-derived scores and MF-derived proportions between AD cases and controls for each cell type. In line with neuronal loss being a hallmark of AD, we observed a lower neuronal score in AD brains compared with controls (Welch’s t test, two-sided, difference in mean score = 0.08, *p* = 0.028; Figure 3C). The same was observed when comparing the MF-derived neuronal proportion in AD cases vs. controls (Welch’s t test, two-sided, difference in mean proportion = 0.13, *p* = 0.001; Figure 3D). We also report a higher oligodendrocyte score (Welch’s t test, two-sided, difference in mean score = 0.07, *p* = 0.013; Figure 3C) but not proportion in AD cases (Welch’s t test, two-sided, difference in mean proportion = 0.06, *p* = 0.15; Figure 3D).

We then used the cell type scores and proportions to re-investigate differential histone acetylation in AD at cell type resolution. Employing the quasi-likelihood *F* test in edgeR,^26^ we first quantified differential acetylation between AD cases and controls, while controlling for CHAS-derived scores and age at death. A total of 5,763 peaks were characterized by hyperacetylation and 5,904 were characterized by hypoacetylation (false discovery rate [FDR] < 0.05; Table S1). To evaluate the likelihood of false-positive associations, we repeated the differential histone acetylation analysis using permuted AD case and control labels. Across 100 permuted datasets, there was never more than one significant peak at FDR <0.05, making it unlikely that the results of our differential acetylation analysis based on the true AD case and control labels were detected due to chance. Differential acetylation analysis was repeated using MF-derived proportions for the four cell types and age at death as covariates. In contrast to controlling for CHAS scores, this analysis revealed a lower number of DARs to the original study,^6^ with 324 hyper- and 792 hypo-acetylated peaks (Table S1). AD-associated hyperacetylated regions from both analyses (controlling for CHAS scores vs. proportions) were significantly enriched for oligodendrocytes when compared with regions that were not differentially acetylated (hypergeometric test, *p* = 4.33 × 10^−57^ when controlling for scores, *p* = 3.25 × 10^−8^ when controlling for proportions; Figure 3E). A similar enrichment of oligodendrocyte- and microglia-specific peaks was observed for AD-associated hypoacetylated regions when controlling for cell type scores (hypergeometric test *p* = 8.87 × 10^−3^ for oligodendrocytes; *p* = 2.48 × 10^−5^ for microglia; Figure 3E). In addition, the top AD-associated hyperacetylated peak when controlling for CHAS scores was specific to oligodendrocytes and located in the vicinity of *MVB12B*, a gene implicated in vesicular trafficking (Figure S5A; Table S1). This peak was also differentially acetylated when controlling for cell type proportions (Table S1). *MVB12B* has previously been identified as an AD risk gene^27^ and forms part of an oligodendrocyte-enriched gene network in the AD brain.^28^ One of the top-ranked AD-hypoacetylated peaks when controlling for CHAS scores was annotated to multiple cell types, and located near *POC1B*, an AD risk gene that was reported to form part of the same core oligodendrocyte gene network as *MVB12B.*^28^ This peak was also differentially acetylated when controlling for cell type proportions (Table S1). To provide a broader overview of the concordance between the analyses controlling for CHAS scores and proportions, we compared the logFC values for acetylation and found a strong correlation (Figure S4A).

Using clusterProfiler,^29^ we were able to match the functional categories associated with AD differentially acetylated bulk peaks to their cell types (Figure 3F). Additionally, we were able to identify cell type-specific dysregulated pathways that were not seen in functional enrichment analyses based on bulk peaks. Neuron-specific hypoacetylated peaks were enriched for synaptic functions (Figure 3F), suggesting an active adaptation of synaptic density and functions in the AD brain, which has been reported by several studies.^30^^,^^31^^,^^32^ As reported previously,^6^ differential H3K27ac was observed in regulatory regions annotated to genes *MAPT*, *APP*, *PSEN1,* and *PSEN2*, which are known to be associated with early-onset AD or directly involved in AD neuropathology (Figure 3G). To link CHAS-annotated cell type-specific acetylation with genetic risk for AD, we performed partitioned heritability analysis.^33^ Supporting previous studies,^4^ significant enrichment of AD risk loci was found within microglia-specific H3K27ac regions but not in the other cell types (Figure 3H).

### Cortical H3K27ac patterns in the PD brain are associated with oligodendrocytes

The role of H3K27ac at the cellular level in PD remains largely unexplored. Although cell type vulnerability in PD is commonly attributed to dopaminergic neurons, genetic risk has been variably associated with cholinergic and enteric neurons, as well as oligodendrocytes.^34^ We applied CHAS to a bulk brain H3K27ac study in PD cases and controls. Toker et al. (2021) observed genome-wide dysregulation of histone acetylation in PFC of individuals with PD from two independent cohorts: the ParkWest (PW) study cohort^35^ and the Netherlands Brain Bank (NBB) cohort (https://www.brainbank.nl/). The authors reported that PD-associated hyperacetylated regions were annotated to genes implicated in PD pathology, and also describe decoupling between promoter H3K27ac and gene expression in the PD brain.^8^ To account for cellular heterogeneity, they integrated H3K27ac differences between NeuN^+^ and NeuN^−^ cell types with brain cell type-specific marker genes and used principal component analysis as a proxy for cell type composition.^8^ This approach revealed no significant differences in cell type proportions between PD cases and controls.

**Figure 4:**
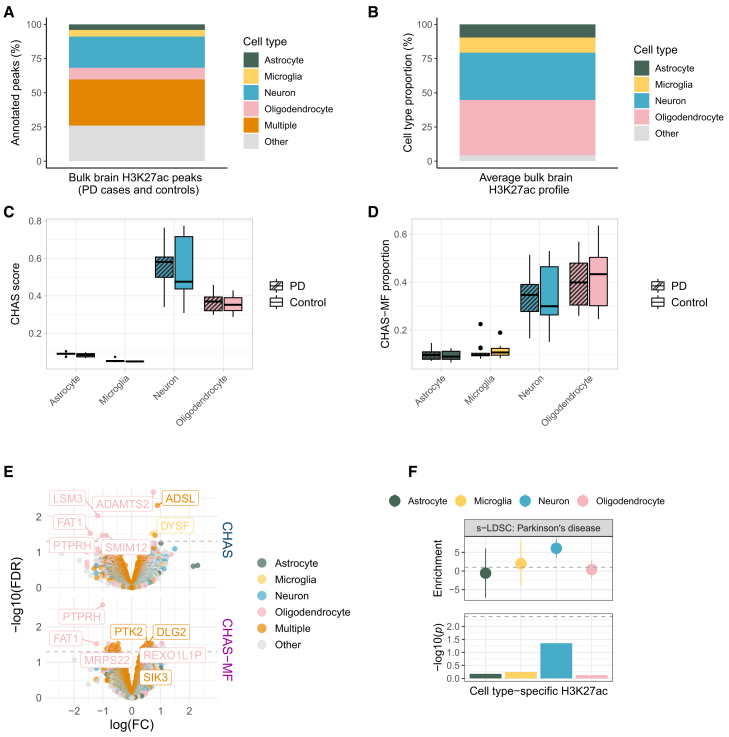
Cortical H3K27ac patterns in the PD brain are associated with oligodendrocytes (A) Stacked barplot of bulk brain H3K27ac peak annotations (PD cases and controls) to astrocytes, microglia, neurons, oligodendrocytes, multiple cell types, or other using CHAS. (B) Stacked barplot of average cell type proportions in bulk brain H3K27ac profiles (PD cases and controls) estimated by CHAS-MF. (C) Boxplot comparing CHAS cell type scores between PD cases and controls (Welch’s t test). (D) Boxplot comparing CHAS-MF cell type proportions between PD cases and controls (Welch’s t test). (E) Volcano plot of DARs between PD cases and controls, controlling for age, sex, and CHAS scores or CHAS-MF proportions. Peaks annotated to genes have FDR <0.05. (F) s-LDSC results using PD GWAS^36^ and CHAS-annotated cell type-specific H3K27ac peaks. Top: LDSC enrichment values. Bottom: −log10(*p*) of coefficient *Z* scores, with Bonferroni significance threshold (dashed line). See also Table S2.

Using the PW cohort (13 PD cases and 10 controls) peaks and read counts generated by Toker et al.,^8^ we filtered out peaks with low read counts and peaks annotated to non-canonical chromosomes before running CHAS. Out of 132,340 peaks, 74% were annotatable to at least one cell type in the PW cohort (Figure 4A). Using CHAS-MF, we estimated the cell type proportion in each bulk sample, and found that the four cell types constituted on average 96% of the samples (Figure 4B). Cell proportions estimated by CHAS-MF from PD and control H3K27ac samples correlated with CHAS scores in all four cell types (Figure S4B). In line with the original study, we found no significant difference in cell type scores or proportions between PD cases and controls (Figures 4C and 4D). While this indicates that bulk PFC tissue may be less prone to confounding disease-associated shifts in cellular proportions in PD, it simultaneously does not represent the primarily disease-affected brain region. Differential histone acetylation analysis controlling for sex, age, and CHAS scores identified four hyperacetylated and three hypoacetylated peaks, of which four were oligodendrocyte-specific and one microglia-specific (Figure 4E). Repeating the analysis using MF proportions instead of CHAS scores revealed 43 peaks characterized by hyperacetylation and 21 peaks characterized by hypoacetylation, and similarly, a large proportion of these peaks were annotated to oligodendrocytes (Figure 4E; Table S2). Common genes were found to be in the vicinity of DARs while controlling for CHAS and CHAS-MF, respectively (Table S2), and logFC values of acetylation between the two analyses were strongly correlated (Figure S4B). Due to the low number of DARs, we chose not to perform cell type or functional enrichment analysis. We also performed partitioned heritability analysis to quantify enrichment of PD risk variants within cell type-specific H3K27ac regions, but did not observe significant enrichment of PD risk in any of the cell types (Figure 4F).

### Cell type deconvolution of autism-associated H3K27ac highlights an epigenetic role for microglia

At the bulk tissue level, dysregulation of H3K27ac in ASD brains is associated with genes involved in synaptic transmission and immunity, as well as genes that harbor rare ASD mutations.^5^ Previously, Sun et al.^5^ performed a histone acetylome-wide association study across three different brain regions from ASD cases and age-matched controls. They reported widespread dysregulation of H3K27ac in PFC and temporal cortex of ASD cases, with similar changes observed in both brain regions. In contrast, only a small proportion of peaks were differentially acetylated in cerebellum.^5^

**Figure 5:**
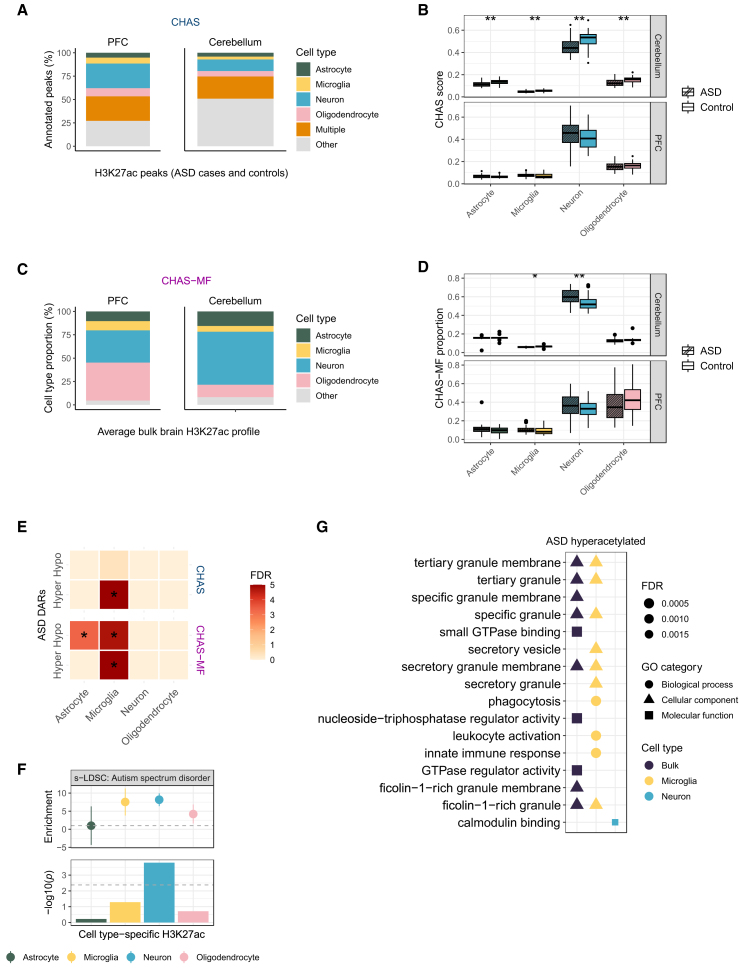
Cell type deconvolution of autism-associated H3K27ac highlights a role for microglia (A) Stacked barplot of bulk PFC and cerebellar H3K27ac peak annotations (ASD cases and controls) to astrocytes, microglia, neurons, oligodendrocytes, multiple cell types, or other using CHAS. (B) Boxplot comparing CHAS cell type scores between ASD cases and controls (Welch's t test, ∗ *p* < 0.05, ∗∗ *p* < 0.01, ∗∗∗ *p* < 0.001, ∗∗∗∗ *p* < 0.0001). (C) Stacked barplot of average cell type proportions in bulk PFC and cerebellar H3K27ac profiles (ASD cases and controls) estimated by CHAS-MF. (D) Boxplot comparing CHAS-MF cell type proportions between ASD cases and controls (Welch’s t test, ∗ *p* < 0.05, ∗∗ *p* < 0.01, ∗∗∗ *p* < 0.001, ∗∗∗∗ *p* < 0.0001). (E) Heatmap of cell type enrichments among ASD-associated DARs, controlling for CHAS scores and CHAS-MF proportions (hypergeometric test, ∗ FDR < 0.05). (F) s-LDSC results using ASD GWAS and CHAS-annotated cell type-specific H3K27ac peaks. Top: LDSC enrichment values. Bottom: −log10(*p*) of coefficient *Z* scores, with Bonferroni significance threshold (dashed line). (G) Functional enrichment analysis of ASD-associated hyperacetylated regions in bulk brain and cell type-specific contexts, showing the top eight enriched pathways per cell type. See also Figure S7 and Table S3.

Using 80 ChIP-seq samples from PFC (40 cases, 40 controls) and 62 samples from cerebellum (31 cases, 31 controls), we called peaks in each brain region using MACS2.^37^ After filtering out peaks with low read counts, we defined an optimal peak set for each brain region: 246,300 peaks in PFC, and 236,358 peaks in cerebellum. We then used these optimal peak sets to run CHAS to evaluate cell type proportions in each brain region, and to generate cell type-specific scores and MF-derived proportions for each sample in each brain region. In the PFC, 73% of bulk peaks were annotatable to at least one cell type, whereas in the cerebellum 49% of peaks could be annotated to a cell type (Figure 5A). This is most likely explained by epigenetic differences across brain regions in the reference and test datasets^38^^,^^39^: brain region-specific differences could exist in the epigenetic state of the same cell type, for instance microglia across multiple brain regions. Similarly, there can be differences in the actual cell types located in different brain regions. For example, the cortex contains highly specialized pyramidal neurons, while purkinje neurons are specific to the cerebellum. Using CHAS-MF, we estimated the cell type proportion in each bulk sample, and found that the four cell types constituted on average 95% of the PFC samples and 92% of the cerebellum samples (Figure 5C).

Next, we compared the cell type scores and proportions in ASD cases with controls, and found no significant difference in either measure (Figures 5B–5D). However, in the cerebellum, ASD cases showed significantly lower cell type scores across all four cell types compared with controls (Figure 5B), likely reflecting inherent cellular composition differences between the cortex and cerebellum. This underscores the importance of using region-specific references. Given these findings, we interpreted ASD-associated differences in cerebellar cell type scores with caution and excluded this dataset from downstream analyses.

Differential histone acetylation analysis controlling for sex, age at death, and cell type score or cell type proportion revealed ASD-associated DARs in PFC (7,652 DARs when controlling for scores, 5,320 DARs when controlling for proportions; Table S3). ASD-associated hyperacetylated regions in PFC, when controlling for cell type scores and proportions, were significantly enriched for microglia when compared with the background peak set (hypergeometric test, *p* < 2.2^−16^; Figure 5E). In addition, cell type enrichment while controlling for cell type proportions revealed astrocyte- and microglia-specific enrichment in ASD-associated hypoacetylated regions (hypergeometric test, *p* = 6.0 × 10^−4^ for astrocyte and *p* = 1.8 × 10^−4^ for microglia; Figure 5E). This is consistent with existing evidence that ASD patients have altered microglial states.^40^^,^^41^ The top ranking microglia-specific ASD-associated hypoacetylated peak was located ∼46 kb upstream of *EMSY* (Figure S3C; Table S3). Transcriptome analysis of cortical samples from ASD cases and controls identified *EMSY* as one of the top downregulated genes and suggested a general role for dysregulated microglial genes.^42^ Moreover, whole exome sequencing in individuals with ASD revealed a *de novo* loss-of-function mutation in this gene.^43^ The top ranking neuron-specific ASD-associated hypoacetylated peak was located 330 base pairs (bp) upstream of *COBLL1*, and was identified among the top five DARs while controlling for both CHAS scores and MF proportions (Figure S3D; Table S3). *COBLL1* was recently identified in a quantitative genome-wide association study using MAGMA to be associated with joint attention and nonverbal communication in ASD patients.^44^ Comparison of the logFC values of acetylation between the two analyses revealed a strong correlation (Figure S4C). We quantified the enrichment of ASD risk variants in cell type-specific H3K27ac and, consistent with previous findings,^4^ found significant enrichment in neuron-specific H3K27ac regions (Figure 5F).

Functional enrichment analysis at the cell type level enabled us to map differentially acetylated bulk peaks to their specific cell types and identify distinct functional enrichments across the four cell types that were not detected in bulk analysis. For example, as reported in the original study,^5^ hyperacetylated peaks in the PFC while controlling for CHAS scores were associated with calmodulin binding (Figure 5G; Table S3). Using CHAS, we additionally report that this enrichment is driven by hyperacetylated peaks that were annotated to neurons (Figure 5G; Table S3). ASD hyperacetylated bulk peaks revealed enrichment for immune-related processes, which were predominantly specific to microglia (Figure 5G; Table S3). This is in line with the growing body of evidence highlighting the role of glial cells and neuroimmune alterations in ASD.^40^^,^^42^^,^^45^^,^^46^^,^^47^

### Deconvolution of bulk PFC H3K27ac in schizophrenia and bipolar disorder reveals neuron- and oligodendrocyte-specific epigenetic dysregulation

Until recently, the epigenomic landscapes of schizophrenia and bipolar disorder remained largely unknown. A landmark study in which 249 bulk brain (PFC) samples (133 controls, 68 schizophrenia cases, and 48 bipolar disorder cases) were profiled for H3K27ac reported genome-wide alterations of this epigenetic mark across the disorders, both at the tissue and neuronal levels.^9^ While the study controlled for oligodendrocytes, glutamatergic neurons, and GABAergic neurons in bulk brain samples, re-analyzing the dataset with cell type-specific signals from astrocytes and microglia could provide additional insights.

**Figure 6:**
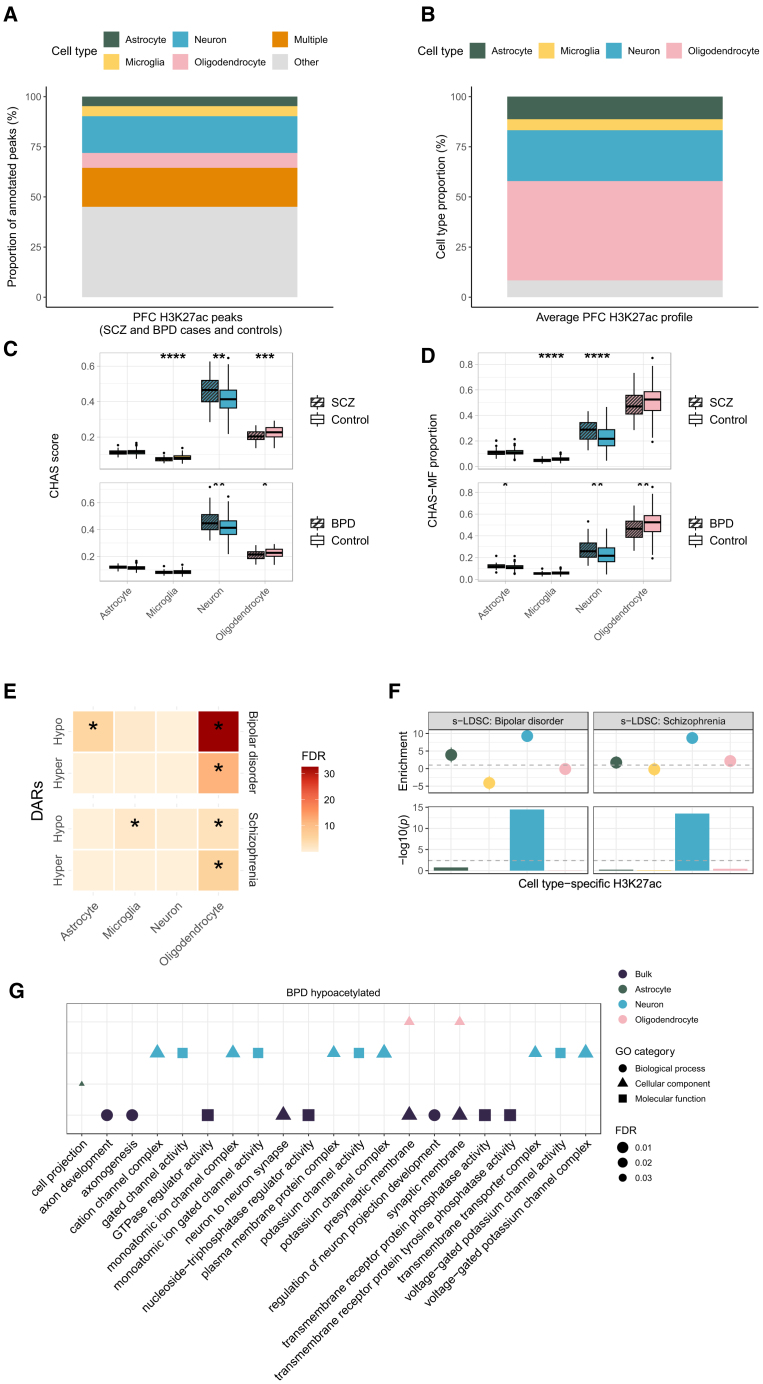
Deconvolution of bulk PFC H3K27ac in schizophrenia and bipolar disorder highlights neuron- and oligodendrocyte-specific epigenetic dysregulation (A) Stacked barplot of bulk PFC H3K27ac peak annotations (schizophrenia cases, bipolar cases, and controls) to astrocytes, microglia, neurons, oligodendrocytes, multiple cell types, or other using CHAS. (B) Stacked barplot of average cell type proportions in bulk PFC H3K27ac profiles (schizophrenia cases, bipolar cases, and controls) estimated by CHAS-MF. (C) Boxplots comparing CHAS cell type scores between schizophrenia cases and controls, and bipolar cases and controls (Welch’s t test, ∗ *p* < 0.05, ∗∗ *p* < 0.01, ∗∗∗ *p* < 0.001, ∗∗∗∗ *p* < 0.0001). (D) Boxplots comparing CHAS-MF cell type proportions between schizophrenia cases and controls, and bipolar cases and controls (Welch’s t test, ∗ *p* < 0.05, ∗∗ p < 0.01, ∗∗∗ *p* < 0.001, ∗∗∗∗ *p* < 0.0001). (E) Heatmaps of cell type enrichments among schizophrenia- and bipolar-associated DARs, controlling for CHAS scores and CHAS-MF proportions (hypergeometric test, ∗ FDR < 0.05). (F) s-LDSC results using schizophrenia and bipolar disorder GWAS and CHAS-annotated cell type-specific H3K27ac peaks. Top: LDSC enrichment values. Bottom: −log10(*p*) of coefficient *Z* scores, with Bonferroni significance threshold (dashed line). (G) Functional enrichment analysis of schizophrenia-associated hypoacetylated regions in bulk brain and cell type-specific contexts, showing the top 10 enriched pathways per cell type. See also Figure S8 and Table S4.

Publicly available FASTQ files for all 249 PFC samples were downloaded and pre-processed before mapping to GRCh38 using Bowtie2.^48^ Peak calling was performed using MACS2^37^ on a merged file of all samples. The bulk PFC H3K27ac peaks were annotated to cell type-specific signals, and cell type scores and proportions were estimated using CHAS and CHAS-MF, respectively. Although both the bulk and reference H3K27ac datasets were from the cortex, only 55% of bulk peaks could be annotated to at least one cell type (Figure 6A). This discrepancy may be due to technical factors, such as the use of fresh resected tissue for reference samples vs. postmortem brain tissue, which can be affected by postmortem interval. Biological differences could also contribute, as the reference data were from young epilepsy patients, while the bulk data included a range of ages and both healthy and diseased brains. Using CHAS-MF, we estimated the cell type proportions in each bulk sample, with the four cell types constituting, on average, 92% of the samples (Figure 6B).

Changes in scores and proportions when comparing cases vs. controls were fairly consistent (Figures 6C and 6D). In the schizophrenia brain, we found a higher neuronal score (*p* = 0.001) and proportion (*p* = 5.5 × 10^−5^) when compared with the controls, and a lower microglial score (*p* = 1.6 × 10^−5^) and proportion (*p* = 1.4 × 10^−6^). In the bipolar disorder brain, we found a higher neuronal score (*p* = 0.004) and proportion (*p* = 0.0064), and a lower oligodendrocyte score (*p* = 0.019) and proportion (*p* = 0.005). Using matched samples, we compared our CHAS-derived oligodendrocyte scores to proportion estimates derived using an independent deconvolution method in the original study and found a significant correlation (Spearman’s rank correlation coefficient, r = 0.95, *p* < 2.2^−16^; Figure S1F).

Differential H3K27ac analysis controlling for age at death, sex, and CHAS scores revealed 235 hyperacetylated and 290 hypoacetylated peaks when comparing schizophrenia cases with controls (FDR <0.05, Table S4). When the scores were swapped for MF proportions as the covariates, this dropped down to 118 hyperacetylated and 102 hypoacetylated peaks (FDR <0.05). Applying the same workflows to the bipolar disorder H3K27ac data, we identified 1,568 hyperacetylated and 1,298 hypoacetylated peaks when controlling for age at death, sex, and CHAS scores (FDR <0.05, Table S4). In contrast, only 159 peaks were differentially acetylated when controlling for MF-derived proportions (FDR <0.05). Given the smaller set of disease-associated peaks identified when controlling for MF proportions, we performed cell type- and pathway enrichment analyses using the results from the analysis when controlling for CHAS scores. For both schizophrenia and bipolar disorder, DARs were most strongly enriched for oligodendrocytes (Figure 6E). One of the top ranking neuron-specific schizophrenia-associated hypoacetylated peaks when controlling for both scores and proportions was located ∼1.4 kb downstream of *VGF* (Figure S3E; Table S4), which has been reported to have differential expression in schizophrenia patients.^49^^,^^50^ In bipolar disorder, we identified common oligodendrocyte-specific differentially acetylated peaks in the vicinity of *AP1S2*, *SIL1*, and *EDAR*, while controlling for CHAS scores and MF proportions, respectively (Figures S3F–S3H; Table S4). Overall, the differential acetylation analysis controlling for cell type scores or proportions were strongly concordant for both H3K27ac datasets (Figures S4D and S4E). Previous gene regulatory studies of psychiatric disorders have largely been neurocentric, whether performed using bulk tissue or neuronal populations. Our results emphasize the significance of studying cell type-specific epigenetic effects, and warrant further investigation of oligodendrocytes in bipolar disorder and schizophrenia. This is particularly relevant in schizophrenia, where transcriptomic and methylomic analyses have highlighted disease-associated changes in this cell type.^19^

Using GWAS for schizophrenia^51^ and bipolar disorder,^52^ we quantified the enrichment of risk SNPs within cell type-specific peaks, and found that the heritability of both psychiatric disorders was exclusively enriched within neuronal-specific H3K27ac peaks (Figure 6F). This is consistent with the original study, in which the authors additionally investigated NeuN^+^ H3K27ac profiles and found that these were most strongly enriched for schizophrenia heritability.^9^ Functional enrichment analysis showed that neuron-specific peaks that were hypoacetylated in bipolar disorder were associated with channel activity and complexes (Figure 6G; Table S4), while hypoacetylated peaks specific to oligodendrocytes were associated with synaptic membrane pathways (Figure 6G; Table S4).

## Discussion

Since histone acetylation is highly cell type specific, deconvolving bulk brain H3K27ac profiles is crucial for interpreting brain disorders. We developed CHAS, a computational tool implementing two independent algorithms for this task. Applied to diverse brain disorders, CHAS scores and CHAS-MF proportions were highly correlated, yielding consistent downstream results.

To illustrate the utility of CHAS for interpretation of bulk tissue H3K27ac profiles, we applied it to five epigenome-wide association studies of brain disorders.^5^^,^^6^^,^^8^^,^^9^ Deconvolution of H3K27ac profiles from the AD brain using both CHAS algorithms highlighted that differential acetylation in late-stage AD is enriched for oligodendrocyte-specific H3K27ac. An independent study also found the largest H3K27ac changes in oligodendrocytes in the hippocampus and dorsolateral PFC of individuals with AD.^53^ Taken together, these data suggest that these oligodendrocyte-specific H3K27ac changes are not limited to a single brain region and warrant further investigation of the role of oligodendrocyte H3K27ac dysregulation in AD. AD-associated hypoacetylation was also observed in microglia, which could reflect an increase in the activity of histone deacetylases (HDACs). In line with this, a recent study found that genetic ablation of microglial HDAC1 and HDAC2 in an AD mouse model reduced amyloid plaque burden and rescued memory deficits,^54^ suggesting HDAC modulation in microglia as a potential therapeutic target. Whereas epigenetic variation in late-stage AD predominantly points to oligodendrocytes, genetic risk enriched in microglial H3K27ac domains, suggesting independent biological mechanisms. This corroborates the finding that genetic risk for AD is enriched at microglia-specific regulatory elements^4^^,^^53^^,^^55^ and genes.^34^ Similarly, for the ASD, bipolar disorder, and schizophrenia datasets, genetic risk was enriched for neuronal-specific H3K27ac, whereas epigenetic dysregulation was most strongly associated with microglia in ASD, and oligodendrocytes in both bipolar disorder and schizophrenia. In the context of epigenetics in ASD, hyperacetylated promoters were linked to upregulated microglial genes,^56^ and differentially methylated regions were shown to be enriched within microglial open chromatin regions.^57^ In addition to highlighting which cell types are epigenetically dysregulated across diverse brain disorders, our analyses guide prioritization of gene and pathway targets in these cell types. For example, the top AD DARs were oligodendrocyte-specific and annotated to genes involved in an oligodendrocyte gene network associated with AD. Taken together, our findings highlight the potential of CHAS in revealing biological insights and aiding prioritization of relevant cell types and pathways for genetic and epigenetic studies of brain disorders.

Going forward, epigenetic studies at single-cell resolution promise to create a more comprehensive picture of the epigenetic landscape associated with neurological and psychiatric disorders. However, as single-cell H3K27ac profiling is still in its early stages of application, generates sparse data, and has not yet been achieved in the human brain, CHAS provides a unique opportunity to infer cell type-specific signatures in bulk brain histone acetylation profiles. Importantly, this can yield insights into epigenetic changes contributing to brain disorder risk and progression that are associated with specific cell types. CHAS is implemented as an open-source R package available at https://github.com/Marzi-lab/CHAS, adding to the existing repertoire of methods for cell type deconvolution.

### Limitations of the study

CHAS’s ability to detect cell type-specific peaks from bulk histone acetylation profiles is linked to the proportion of each cell type in the bulk tissue sample. This affects the robustness of cell type-specific scores and proportions, as well as the detection of differential acetylation in low-frequency cell types. Including additional covariates, such as cell type-specific scores or proportions, may reduce power in smaller studies. However, in moderate and large studies, like the ones reported here on AD and ASD, controlling for cell type proportions may improve power by reducing noise from cellular heterogeneity. The cell-sorted data on which CHAS is based has three limitations: It includes only four major brain cell types from the cortex, excluding rarer cell types like pericytes and endothelial cells; it lacks diversity in cell subtypes, limiting the ability to distinguish between functionally and regionally distinct neuron types; and it is limited with regard to different cell states. For example, multiple microglial phenotypes have been identified transcriptionally and functionally.^58^^,^^59^^,^^60^^,^^61^ Our current reference is based on a neuropathology-free, pediatric dataset in which such states, if present, are aggregated into one category. Additionally, the performance of CHAS in bulk cerebellum samples underscores the need for brain-region-specific reference datasets to capture regional differences in cell types and states.

At present, CHAS is limited to bulk brain studies of H3K27ac because of reference atlas availability. However, CHAS can be easily extended to other brain cell types, regions, or completely distinct tissues, as well as different histone modifications, with appropriate reference datasets. Most promisingly, we hope that future availability of single-cell H3K27ac profiles across brain regions will enable us to adapt CHAS to deconvolute more refined cell subtypes and states. Finally, concerns have been raised regarding the extent to which given cell deconvolution methods can appropriately account for cellular heterogeneity, particularly when a specific trait is linked to a cellular change such as neuronal loss in AD.^15^ It is reassuring to observe AD-associated epigenetic dysregulation enriched in oligodendrocytes, but not neurons, consistent with findings from independent epigenomic and transcriptomic studies in both humans and mice.^13^^,^^62^

## Resource availability

### Lead contact

Requests for further information should be directed to the lead contact, Sarah J. Marzi (sarah.marzi@kcl.ac.uk).

### Materials availability

This study did not generate new unique reagents.

### Data and code availability

• All supplementary tables are available at: https://doi.org/10.5281/zenodo.12784761.
• CHAS is an open-source R package: https://github.com/Marzi-Lab/CHAS. All the data and code required to reproduce the figures in this manuscript are available at: https://github.com/Marzi-lab/CHAS_manuscript and Zenodo.^63^
• Any additional information required to reanalyze the data reported in this paper is available from the lead contact upon request.

## Acknowledgments

S.J.M. and A.N. are supported by the Edmond and Lily Safra Early Career Fellowship Program and the 10.13039/501100017510UK Dementia Research Institute (award number UKDRI-6009 and UKDRI-5016) through UK DRI Ltd, principally funded by the 10.13039/501100000265Medical Research Council. S.J.M. received funding from the 10.13039/100000957Alzheimer's Association (grant number ADSF-21-829660-C) and the 10.13039/501100000265MRC (grant number MR/W004984/1). K.B.M. was funded by the UK Medical Research Council Doctoral Training Partnership (https://mrc.ukri.org). We thank Alan E. Murphy and Brian M. Schilder at the UK Dementia Research Institute, Imperial College London, for feedback and helpful discussions on the CHAS R package. The graphical abstract was created in Biorender: Marzi, S. (2025), https://BioRender.com/qo8nobi.

## Author contributions

Conceptualization, S.J.M.; methodology, S.J.M.; software, K.B.M. and Y.Y.; formal analysis, K.B.M. and Y.Y.; validation, K.B.M. and Y.Y.; validation, K.B.M. and Y.Y.; investigation, A.N. and S.J.M.; data curation, K.B.M., S.J.M., A.N., and M.T.; writing – original draft, K.B.M. and S.J.M.; writing – reviewing & editing, K.B.M., S.J.M., A.N., M.T., and Y.Y.; visualization, K.B.M. and Y.Y.; supervision, S.J.M.; project administration, S.J.M.

## Declaration of interests

The authors declare no competing interests.

## STAR★Methods

### Key resources table

**Table:** 

REAGENT or RESOURCE	SOURCE	IDENTIFIER
**Deposited data**		

Human Alzheimer’s disease ChIP-seq H3K27ac 2018	Marzi et al., 2018^7^	PRJNCA297982
Human Parkinson’s disease ChIP-seq H3K27ac 2021	Toker et al., 2021^9^	https://github.com/ltoker/ChIPseqPD
Human Autism spectrum disorder ChIP-seq H3K27ac 2016	Sun et al., 2016^6^	syn4587616
Human Schizophrenia and Bipolar disorder ChIP-seq H3K27ac 2022	Girdhar et al., 2022^10^	syn25705564
Human NeuN^+^ and NeuN^−^ ChIP-seq 2018	Girdhar et al., 2018^15^	syn4566010
Human astrocyte, microglia, neuron, and oligodendrocyte ChIP-seq H3K27ac 2019	Nott et al., 2018^5^	phs001373.v2.p1

**Software and algorithms**		

CHAS R package	This paper	https://github.com/Marzi-lab/CHAS and https://doi.org/10.5281/zenodo.15120736
FASTQC	Andrews 2010^64^	RRID: SCR_014583
Bowtie2	Langmead and Salzberg 2012^55^	RRID: SCR_016368
Picard tools MarkDuplicaes.jar	https://broadinstitute.github.io/picard/	RRID: SCR_006525
SAMtools	Li et al., 2009^25^	RRID: SCR_002105
MACS	Zhang et al., 2008^44^	RRID: SCR_013291
featureCounts	Liao et al., 2014^65^	RRID: SCR_012919
edgeR	Robinson et al., 2010^28^	RRID: SCR_012802
LDSC	Finucane et al., 2015^39^	RRID: SCR_022801
clusterProfiler	Yu et al., 2012^32^	RRID: SCR_016884

### Method details

#### CHAS cell type scores

CHAS is split into two independent algorithms. In the first, cell type-specific H3K27ac annotation is based on H3K27ac profiles from purified populations of astrocytes, microglia, neurons, and oligodendrocytes.^4^ For a given human bulk brain H3K27ac dataset, CHAS overlaps peaks detected in bulk with each cell type peak set to identify cell type-specific peaks within the bulk H3K27ac profiles. No specific overlap threshold is required in this step; even a single base pair overlap is sufficient for annotation. To derive cell type-specific scores, only high-confidence, highly cell type-specific peaks are included. Two criteria must be met: (i) the bulk peak must be annotated to a single cell type, and (ii) the bulk peak must overlap a predefined percentage of the given cell type peak. This percentage can be specified in the *CelltypeSpecificPeaks()* function, with a default value of 50%.

For each sample, CHAS generates a cell type-specific score by averaging the normalised signal intensity across all peaks specific to the cell type. First, read counts across peaks are converted to CPM to account for library size variation. The signal intensity for each peak is then normalised by dividing the counts by the highest observed read count for that peak, scaling the peak-normalised counts between 0 and 1. For a sample (y) and cell type (x), the peak-normalised counts (sp,y) are summed across all peaks specific to cell type x and divided by the total number of cell type-specific peaks for cell type x (Px). Each score is further normalised by the number of cell type-specific peaks. This ensures that cell types with more specific peaks receive a higher CHAS score. As a result, CHAS scores range from 0 to 1 for any given sample and cell type.

**Figure:**
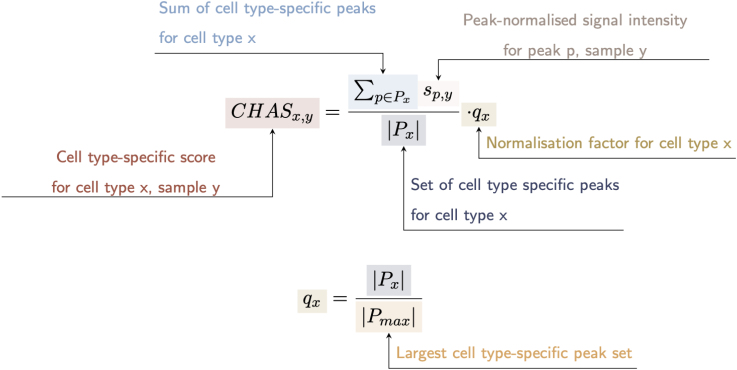

#### CHAS matrix factorization

The second algorithm in CHAS uses a non-negative matrix factorization approach, based on the EPIC R package.^66^ This method models bulk counts as the sum of cell type-specific counts weighted by the corresponding cell type proportion. The bulk count matrix is represented as the product of the reference count matrix and the cell proportion estimation matrix. EPIC then estimates the cell proportions by solving this equation using constrained least square optimization.^66^

**Figure:**
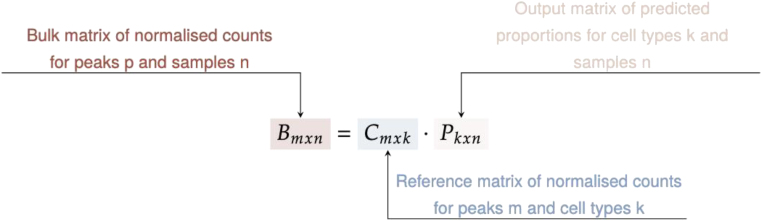

Since EPIC was originally designed for transcriptomic data, we modified it to improve accuracy for deconvolving bulk H3K27ac profiles. Before running the deconvolution, bulk and reference H3K27ac counts are normalised for peak length and library size, similar to RNA-seq normalization. This involves dividing the raw count by the peak’s base pair length, then converting it to a CPM matrix using EdgeR, analogous to the TPM normalisation in EPIC (formula 2, EPIC manuscript^20^).

Second, if multiple reference samples are available for a cell type, the median normalised CPM is used for deconvolution. To account for signal variability, peaks are assigned weights for matrix factorization, prioritizing those with low variability. The variability metric is derived as in the original EPIC manuscript^20^ (formula 7), using half the CPM range across samples.

Third, instead of running matrix factorisation on all consensus peaks, CHAS-MF selects a set of signature peaks with cell type specific signals. These are peaks that have high acetylation signals in one cell type and low signals in all the other cell types. As a threshold to qualify as signature peak, the normalised CPM for the cell type with the highest signal must be at least five times the signal from any other cell type.

The first step in CHAS-MF is merging bulk peaks and reference peaks to create consensus peaks - which are defined as the union of bulk and reference peaks. Each consensus peak is then annotated with corresponding cell types, depending on whether the consensus peak overlaps cell type-specific peaks. Read counts are generated for the consensus peaks, either from bam files or existing counts for the bulk and reference samples. Using this count data, CHAS-MF predicts the proportion of each cell type in bulk samples by applying the EPIC R package, using our histone acetylation optimized adaptations. The calculation is performed on normalised read counts for signature peaks, controlling for library size and peak lengths. If multiple reference samples are used for one cell type, the median normalised CPM will be used as the main input for matrix factorisation, and the signal variability for each peak is taken into account as a weight term in the matrix factorisation.

The cell type annotation, generation of cell type-specific histone acetylation scores, and generation of MF-derived cell type proportion estimates are implemented and automated in our R package CHAS (https://github.com/Marzi-lab/CHAS).

#### Validation of CHAS

We validated CHAS using three independent approaches. First, by simulating pseudobulk H3K27ac profiles of known cell type composition based on the raw sequencing data from astrocytes, microglia, neurons and oligodendrocytes by Nott and colleagues.^4^ The cell type composition of each pseudobulk sample was based on proportions of astrocytes, microglia, neurons, oligodendrocytes and endothelial cells in brain tissue from older individuals, which had been quantified in an independent study using immunohistochemistry.^21^ Based on the reported cell type proportions of 49 postmortem brain samples we generated 49 pseudobulk samples, pooling a total of 30 million randomly sampled reads per sample from the raw H3K27ac data of the four cell types. As our reference did not include H3K27ac profiles for endothelial cells, we excluded the proportion of this cell type and instead used the relative proportions of the four other cell types. We ran CHAS and CHAS-MF to generate cell type-specific scores and cell type proportions, respectively, for each pseudobulk sample and compared these to the true cell type proportions using Spearman’s rank correlation coefficients. To additionally evaluate the robustness of CHAS with respect to sample size and sequencing depth, this process was repeated across the 49 samples with 20 million and 10 million randomly sub-sampled reads, as well as using 30 million reads in random subsets of 25 and 10 samples. Second, we used CHAS with an existing dataset of NeuN^+^ and NeuN^−^ H3K27ac peaks from the anterior cingulate cortex (*n* = 15) and dorsolateral PFC (*n* = 14). The *CelltypeSpecificPeaks()* function was used to annotate each peak set, and then using the peaks that were annotatable to at least one cell type, we calculated the CHAS scores for each cell type. We then used a paired t-test to compare the average neuronal proportion across the NeuN^+^ peaks with that of the NeuN^−^ peaks. As the CHAS-MF workflow requires H3K27ac read counts, the associated NeuN^+^ and NeuN^−^ bam files were downloaded and pre-processed in the same way as was done for the AD H3K27ac dataset (see STAR Methods: H3K27ac in entorhinal cortex from AD cases and controls (Marzi et al. 2018)). Consensus peaks were identified using the *ConsensusPeaks()* function on the processed peaks and counts data. The cell type proportion was estimated using the *CelltypeProportion()* function. We repeated the t-test comparing the average MF-derived neuronal proportion of NeuN^+^ peaks with that of the NeuN^−^ peaks. Finally, for the AD^6^ and schizophrenia and bipolar disorder^9^ datasets, we were able to correlate CHAS-derived neuronal and oligodendrocyte scores, respectively, with cellular proportions estimated independently in the original studies. In the AD H3K27ac study, neuronal proportions were quantified based on methylation profiles using CETS,^10^ and in the schizophrenia and bipolar disorder H3K27ac study, oligodendrocyte proportions were quantified based on acetylation profiles using dtangle.^67^

#### Application of CHAS to bulk brain H3K27ac datasets

##### H3K27ac in entorhinal cortex from AD cases and controls (Marzi et al. 2018)

In order to demonstrate reproducibility and undertake preprocessing using updated versions of software and the most recent reference genome, raw ChIP-seq data from our previous study was downloaded from sequence read archive (SRA) under accession number PRJNCA297982.^6^ We performed basic quality control using fastQC.^68^ Using bowtie2^48^ the fastq files were aligned to the most recent human reference genome (GRCh38).^69^ The resulting SAM files were converted to binary (BAM) format using SAMtools.^70^ Duplicates, unmapped reads, and reads with a sequence quality score q < 30 were removed from all BAM files and the filtered BAM files were subsequently merged into one grouped file. Next, using MACS2^37^ we performed peak calling on the merged file of all samples. The following peak sets were subsequently filtered out: 1) peaks which overlapped the ENCODE blacklist peaks (https://github.com/Boyle-Lab/Blacklist), 2) peaks which were located in unmapped contigs or mitochondrial DNA, and 3) peaks which did not meet a significance threshold of *p* < 10^−7^ for peak calling. Read count generation for each sample was performed using featureCounts^71^ and read counts were converted to and stored in a CPM matrix, keeping peaks with a minimum of three samples showing ≥1 read per million. This resulted in a total of 183,353 peaks to be used in downstream analyses. This optimal peak set and CPM matrix were used as input to run CHAS to identify cell type-specific peaks in the bulk H3K27ac profiles and to generate cell type-specific H3K27ac scores as a proxy for the proportion of each cell type in the bulk peak set. Of note, to annotate cell types to each bulk peak and to calculate the cell type proportions across the bulk peaks, we only required an overlap of at least one base pair between the bulk peak and the cell type peak. However, for peaks included in the cell type-specific histone acetylation score we required a more stringent overlap of at least 50% of the cell type peak interval. We also ran CHAS-MF on the processed peaks and counts along with the BAM files to estimate the proportions of each cell type in the bulk samples. The CHAS-generated cell type-specific scores and CHAS-MF-derived proportions were used to detect shifts in cellular composition between AD cases and controls, by comparing the means using Welch’s t-test. Differences in histone acetylation between AD cases and controls were analyzed as previously described,^6^ but including the CHAS derived cell type scores or MF proportions, instead of the neuronal proportion estimator based on CETS.^10^ Briefly, the quasi-likelihood F test in the Bioconductor package edgeR^26^ was used to test for differences in histone acetylation between AD cases and controls, while controlling for: (i) age at death and cell type-specific scores for the four brain cell types; (ii) age at death and cell type proportions. All covariates were treated as continuous numeric variables. Peaks were considered differentially acetylated at FDR <0.05. To additionally confirm that we had adequately controlled for false-positive associations, we permuted the AD case and control labels 100 times and repeated the differential histone acetylation analysis as described above.

##### H3K27ac in PFC from PD cases and controls: Toker et al. 2021

Peak lists and read count tables for the Park West (PW) cohort were downloaded from https://github.com/ltoker/ChIPseqPD. Peak lists were in narrowPeak format and were filtered to include peaks mapping to canonical chromosomes, and to exclude peaks which overlapped those in blacklisted regions https://github.com/Boyle-Lab/Blacklist, as well as those not meeting a significance threshold of *p* < 10^−7^ for peak calling. For the PW cohort, a total of 171,285 peaks were used for downstream analyses. From the counts tables we excluded the sample outliers identified in Toker et al. (2021) and performed final filtering, keeping peaks with a minimum of three samples showing ≥1 read per million for the differential histone acetylation analysis. This left us with 152,823 peaks in the PW cohort. The counts table along with the filtered peak set were used as input to CHAS and CHAS-MF, as previously described for the AD dataset. As there were no BAM files available for the PD study, we performed CHAS-MF using the original counts for the bulk and reference peaks as a proxy for the read counts for consensus peaks. The CHAS-generated cell type-specific scores and CHAS-MF-derived proportions were used to detect shifts in cellular composition between PD cases and controls, by comparing the means using Welch’s t-test. Differences in histone acetylation between PD cases and controls were analyzed as described above, controlling for age at death, sex, and cell type proportions using the: (i) CHAS-derived cell type scores, or (ii) CHAS-MF proportions.

##### H3K27ac in PFC and cerebellum from ASD cases and controls: Sun et al. 2016

ChIP-seq reads mapped to the human reference genome (hg19) using BWA^64^ by Sun and colleagues (2016) were downloaded from Synapse under accession number syn4587616. We downloaded 80 libraries from the PFC and 62 libraries from the cerebellum. These were the same libraries that were used in the original study for peak calling,^5^ with exception of one PFC sample which was not available on Synapse. Downloaded files were in BAM format and all pre-processing steps were performed as described previously (see H3K27ac in entorhinal cortex from AD cases and controls (Marzi et al. 2018) under STAR Methods). The optimal peak sets for downstream analyses totaled 250,614 peaks for PFC, and 241,759 peaks for cerebellum. These, alongside the counts matrices, were used as input to CHAS. We also ran CHAS-MF on the same peaks and counts data, along with the BAM files, to estimate the proportion of each cell type in the bulk samples. Differences in histone acetylation between ASD cases and controls for each brain region were analyzed as described above, controlling for age at death, sex, and: (i) CHAS-derived cell type scores, (ii) CHAS-MF proportions. Disease-associated differences in cell type proportions were quantified using a Welch’s t-test on the CHAS-derived cell type-specific scores and the CHAS-MF derived cell type proportions.

##### H3K27ac in PFC from schizophrenia and bipolar disorder cases and controls: Girdhar et al. 2022

Raw sequencing data (FASTQ files) for 249 samples were downloaded from Synapse under accession number syn25705564. Of these libraries, 68 were from schizophrenia cases, 48 were from bipolar disorder cases, and 133 were from controls. All pre-processing, mapping, and peak calling steps were performed as described previously (see H3K27ac in entorhinal cortex from AD cases and controls (Marzi et al. 2018), under STAR Methods), with peaks being called on a merged file containing all schizophrenia, bipolar disorder, and control samples. 396,065 peaks were used as input to CHAS and CHAS-MF, as well as for differential H3K27ac analysis controlling for sex, age at death, and: (i) cell type-specific histone acetylation scores, (ii) cell type proportions. Disease-associated differences in cell type proportions were quantified using a Welch’s t-test on the CHAS-derived cell type-specific scores and the CHAS-MF derived cell type proportions.

##### Quantifying enrichments of cell type-specific H3K27ac peaks within disease-associated differentially acetylated regions

To test whether the proportion of cell type-specific peaks in the disease-associated DARs differed significantly from the background set of cell type-specific non-DARs, we used the hypergeometric test. *p* values were corrected for multiple testing using an FDR cutoff of 0.05. This was calculated for the AD, ASD, bipolar disorder, and schizophrenia H3K27ac peaks.

##### Genomic annotation and enrichment analysis

Gene annotation and gene ontology analyses were performed using clusterProfiler.^29^ Gene annotation was performed using the *annotatePeak()* function using the default parameters and additionally specifying *annoDb=“org.Hs.*e.g.,*.db”* to retrieve gene symbols. Gene ontology analysis was performed using the *enrichGO()* function, for the ontology categories biological process, molecular function, and cellular component. Enrichment *p* values were corrected for multiple testing using an FDR cutoff of 0.05.

##### Partitioned heritability analysis

To estimate the proportion of disease SNP-heritability attributable to cell type-specific H3K27ac peaks identified in bulk brain data, we performed partitioned heritability analysis as implemented in LDSC.^33^ For each cell type-specific peak set, annotation files were generated and used to compute LD scores. Publicly available GWAS summary statistics for an AD GWAS,^25^ ASD GWAS,^65^ PD GWAS,^36^ schizophrenia GWAS,^51^ and bipolar disorder GWAS,^52^ were downloaded and converted to the required format for LDSC. Steps for the analysis were followed as instructed here https://github.com/bulik/ldsc/wiki. For each annotation, LDSC was run using the full baseline model,^33^ thereby computing the proportion of SNP-heritability associated with the annotation of interest, while taking into account all the annotations in the baseline model.

## Supplemental information

Document S1. Figures S1–S8

Table S1. Alzheimer’s disease H3K27ac CHAS analysis, related to Figure 3

Table S2. Parkinson’s disease H3K27ac CHAS analysis, related to Figure 4

Table S3. Autism spectrum disorder H3K27ac CHAS analysis, related to Figure 5

Table S4. Schizophrenia and bipolar disorder H3K27ac CHAS analysis, related to Figure 6

Document S2. Article plus supplemental information
