# Supplementary material for: CHAS infers cell type-specific signatures in bulk brain histone acetylation studies of neurological and psychiatric disorders
Source: Cell Rep Methods. 2025 Apr 28;5(5):101032. doi: 10.1016/j.crmeth.2025.101032 (PMC12146652; doi:10.1016/j.crmeth.2025.101032)
Supplement: Document S1. Figures S1–S8 [file mmc1.pdf]

Cell Reports Methods, Volume *5*

Supplemental information

CHAS infers cell type-specific signatures in bulk brain histone acetylation studies of neurological and psychiatric disorders

Kitty B. Murphy, Yuqian Ye, Maria Tsalenchuk, Alexi Nott, and Sarah J. Marzi

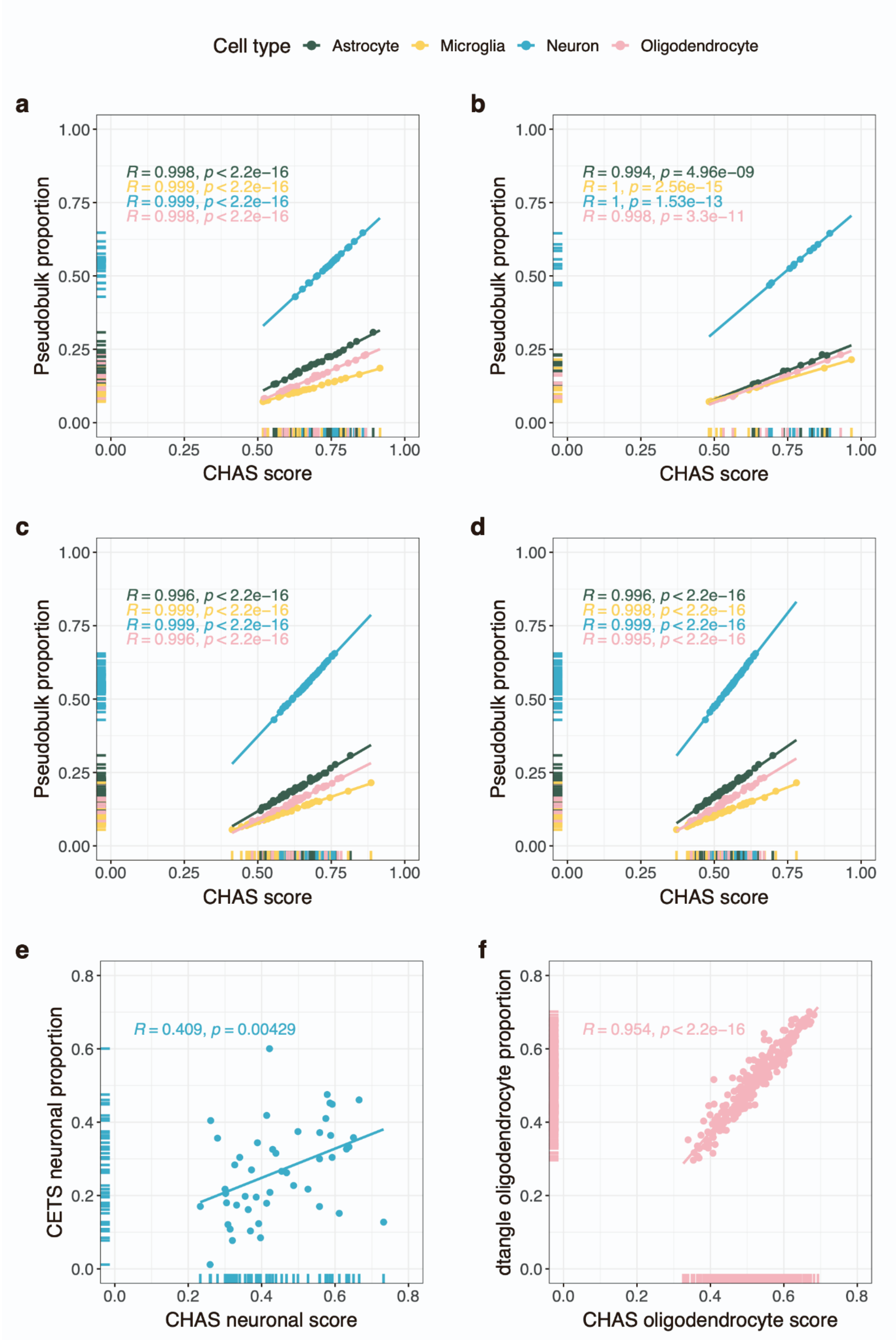

Supplementary Figure 1, related to Figure 2: Validation of CHAS using pseudobulk samples and independent estimates. a–d Scatterplots compare CHAS-derived histone acetylation scores (x-axis) with true cell type proportions (y-axis) in pseudobulk samples composed of randomly sampled reads from astrocytes, microglia, neurons, and oligodendrocytes. Analyses were performed across different sample sizes and read depths: **a** 25 samples (30M reads), **b** 10 samples (30M reads), **c** 49 samples (20M reads), and **d** 49 samples (10M reads). **e–f** CHAS scores were further validated against independent cell proportion estimates. **e** CHAS-derived neuronal scores significantly correlated with CETS-derived NeuN^{+} fractions (R = 0.41, p = 0.004) across 47 AD patient and control samples, based on DNA methylation profiles from the entorhinal cortex. **f** CHAS-derived oligodendrocyte scores showed a strong correlation with dtangle-estimated oligodendrocyte proportions (R = 0.95, p < 2.2 × 10^{-16}) in 249 schizophrenia and bipolar disorder samples.

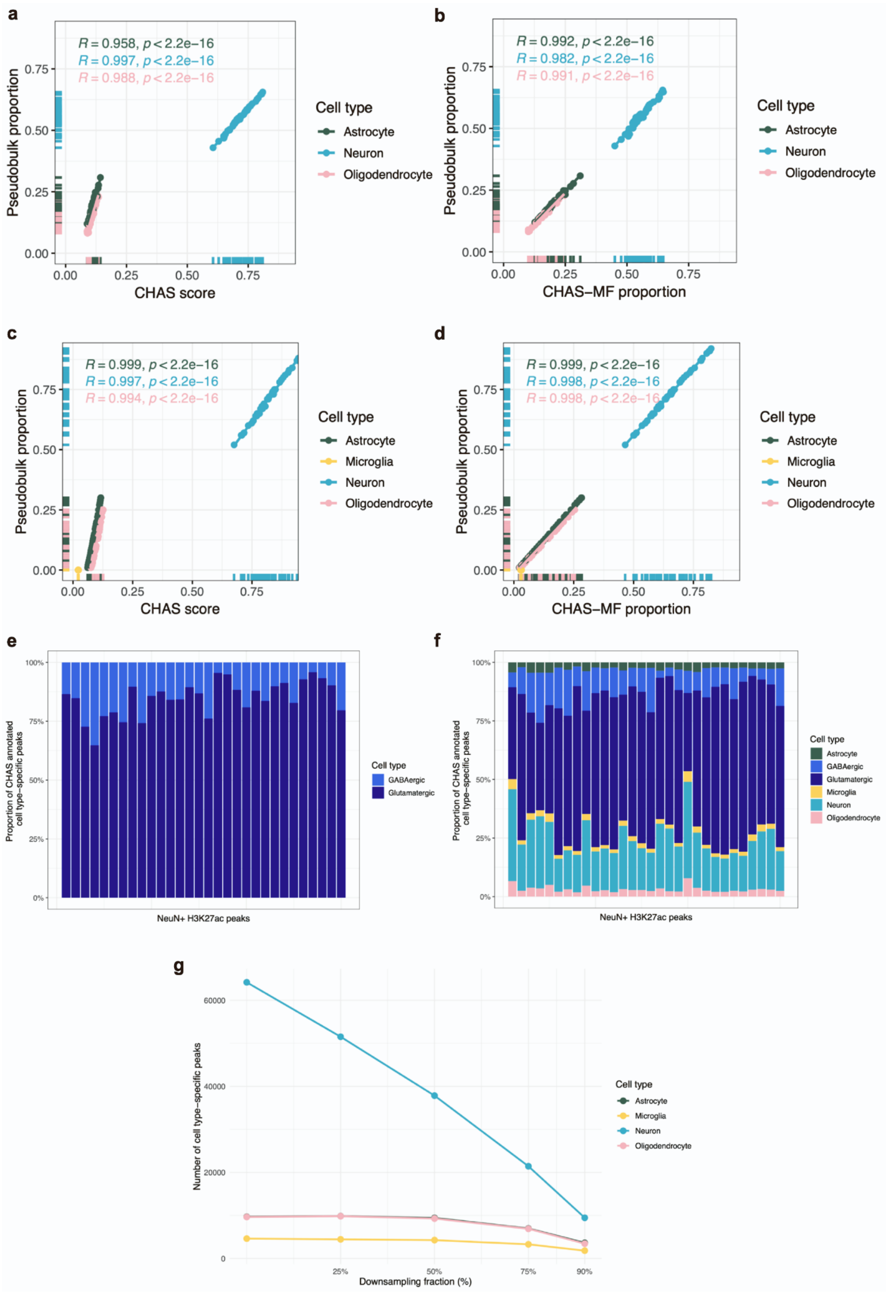

Supplementary Figure 2, related to Figure 2: Evaluating CHAS in different contexts. Scatterplots of the pseudobulk proportion for 49 samples vs. **a** the CHAS-derived histone acetylation score (x-axis) and **b** the CHAS-derived proportion (x-axis). To simulate the presence of a new cell type in a bulk sample, the microglia reference data was removed from CHAS. Scatterplots of the pseudobulk proportion for 46 samples vs **c** the CHAS-derived histone acetylation score (x-axis) and **d** the CHAS-derived proportion (x-axis). To simulate the absence of a cell type from a bulk sample, pseudobulk samples made up of 30 million randomly sampled reads from astrocytes, neurons, and oligodendrocytes were generated. Spearman's rank correlation coefficient R and P values are shown for each individual cell type. Barplot showing the proportion of annotated cell type-specific peaks in bulk $NeuN^+$ samples using **e** glutamatergic and GABAergic H3K27ac profiles[5], and **f** glutamatergic and GABAergic H3K27ac profiles and the cell type reference data included in CHAS. **g** Scatterplot showing the number of cell type-specific peaks that CHAS is able to resolve within the bulk sample with progressive downsampling of the cell type reference peaks.

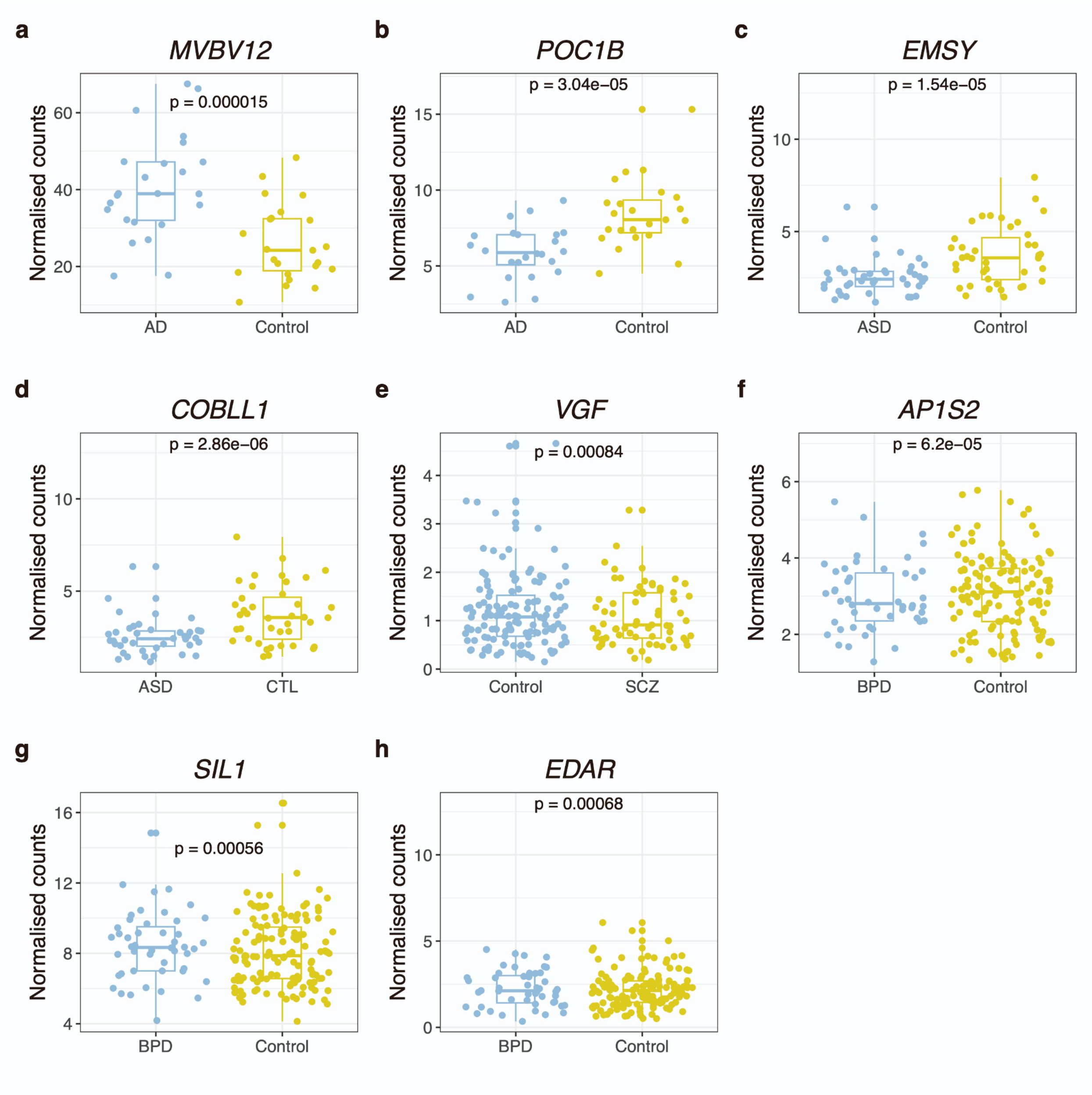

Supplementary Figure 3, related to Figures 3, 4, 5 and 6: Normalised counts for top differentially acetylated peaks and their annotated genes in cases versus controls. Peaks were annotated to genes using the *annoatatePeak()* function in ChIPseeker[6]. P values were obtained from the differential acetylation analysis controlling for CHAS scores using edgeR and were corrected using the FDR.

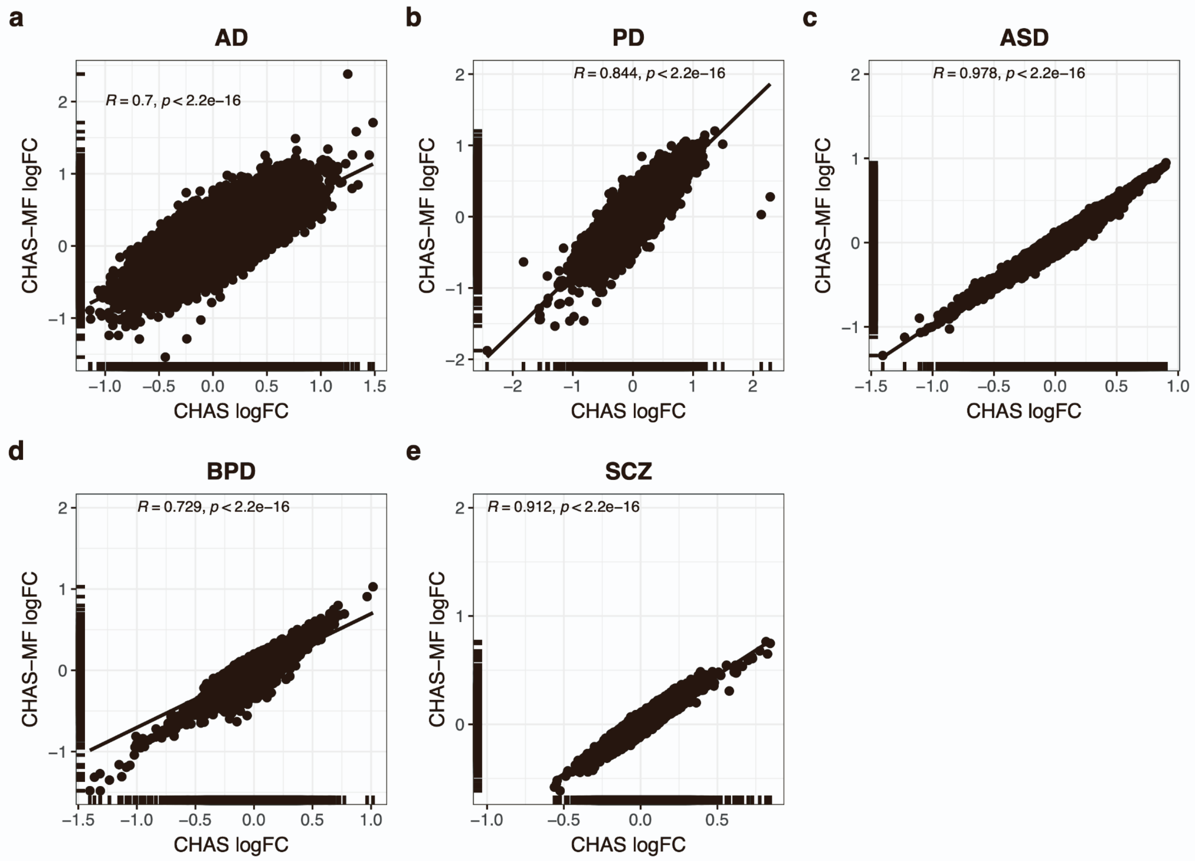

Supplementary Figure 4, related to Figures 3, 4, 5 and 6: Acetylation changes quantified whilst controlling for cell type scores and CHAS-MF derived cell type proportions correlate strongly. Scatterplots of logFC when performing differential acetylation analysis whilst controlling for CHAS-derived scores vs CHAS-MF-derived cell type proportions in the **a** AD H3K27ac dataset[2]**, b** PD H3K27ac dataset[7]**, c** ASD H3K27ac dataset[8]**, d,e** BPD and SCZ H3K27ac dataset[4]**.** Spearman's rank correlation coefficient *R* and *p*-values are shown for each dataset. .

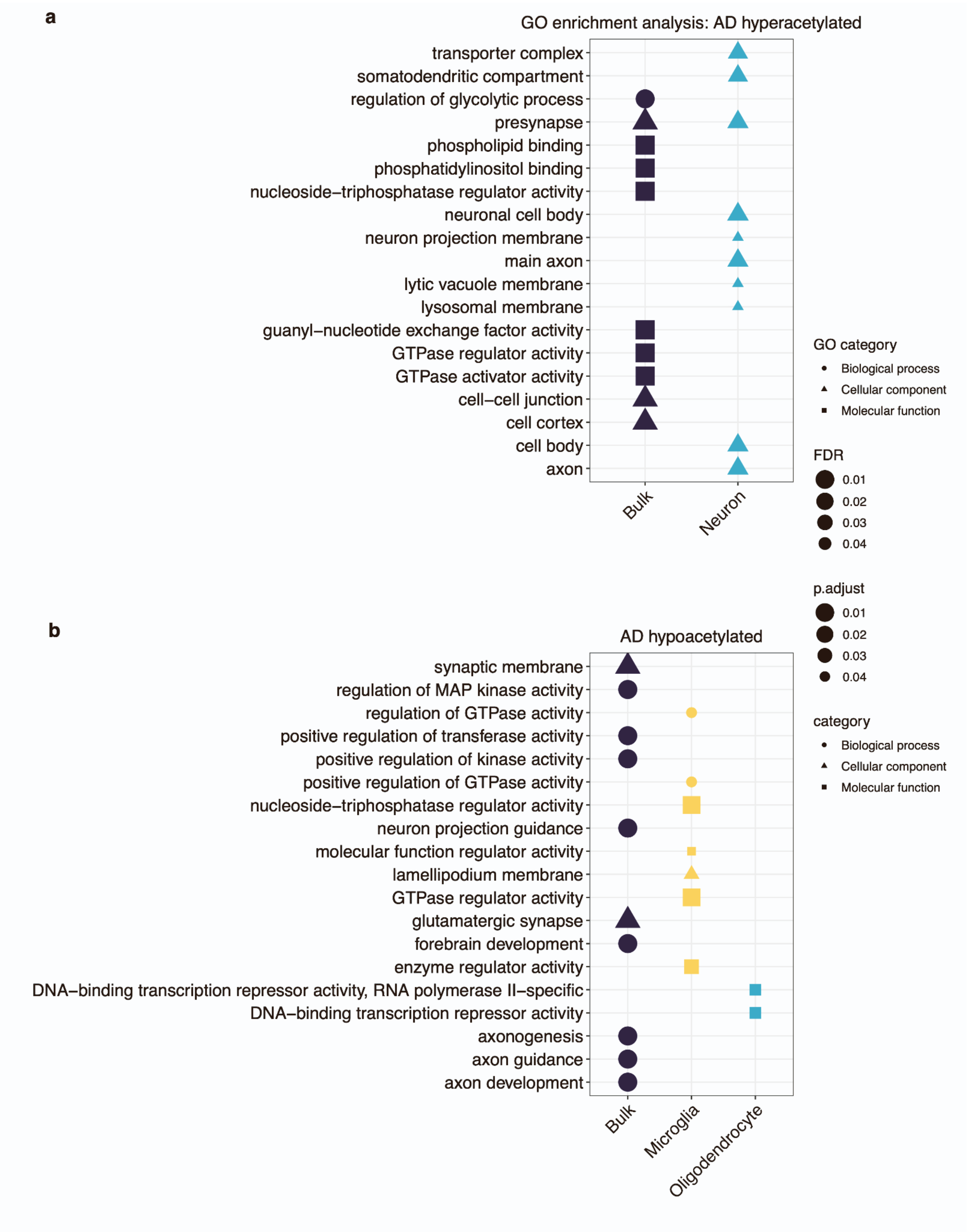

Supplementary Figure 5, related to Figure 3: Functional enrichment analysis using AD-associated H3K27ac regions. Pathway enrichment analysis using AD-associated bulk and cell type-specific **a** hyperacetylated peaks controlling for CHAS scores, (**b**) hypoacetylated peaks controlling for MF-derived proportions. Shown are the top 10 enriched pathways for each cell type. P values were corrected using FDR.

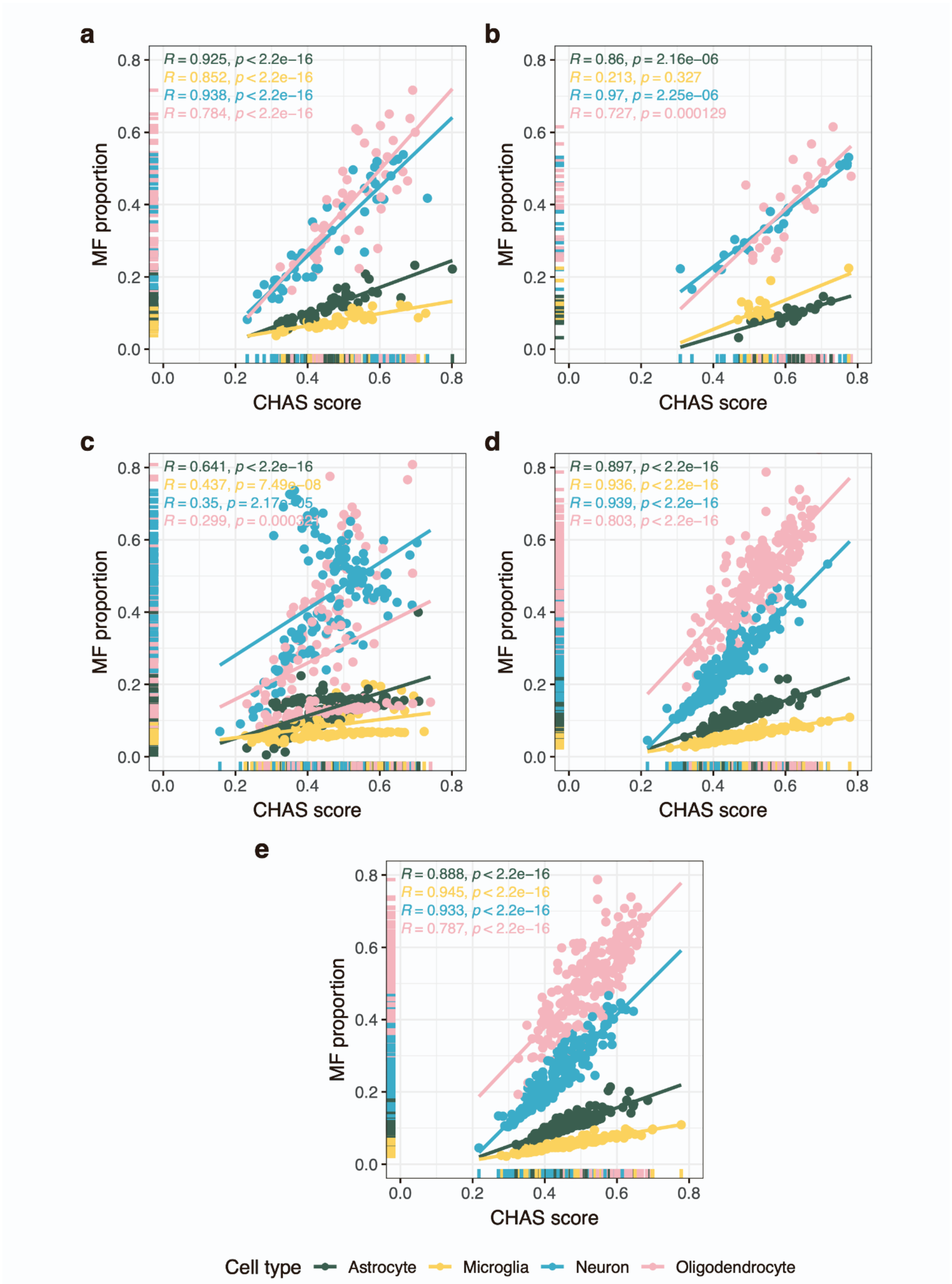

Supplementary Figure 6, related to Figures 3, 4, 5 and 6: Correlations of CHAS scores and CHAS-MF proportions for each brain disorder H3K27ac dataset. a Scatterplot of CHAS-derived scores vs. CHAS-MF-derived proportions for the AD H3K27ac dataset. **b** Scatterplot of CHAS-derived scores vs. CHAS-MF-derived proportions for the PD H3K27ac dataset. **c** Scatterplot of CHAS-derived scores vs. CHAS-MF-derived proportions for the ASD H3K27ac dataset. **d** Scatterplot of CHAS-derived scores vs. CHAS-MF-derived proportions for the BPD H3K27ac dataset. **e** Scatterplot of CHAS-derived scores vs. CHAS-MF-derived proportions for the SCZ H3K27ac dataset. Spearman's rank correlation coefficient *R* and *p*-values are shown for each cell type.

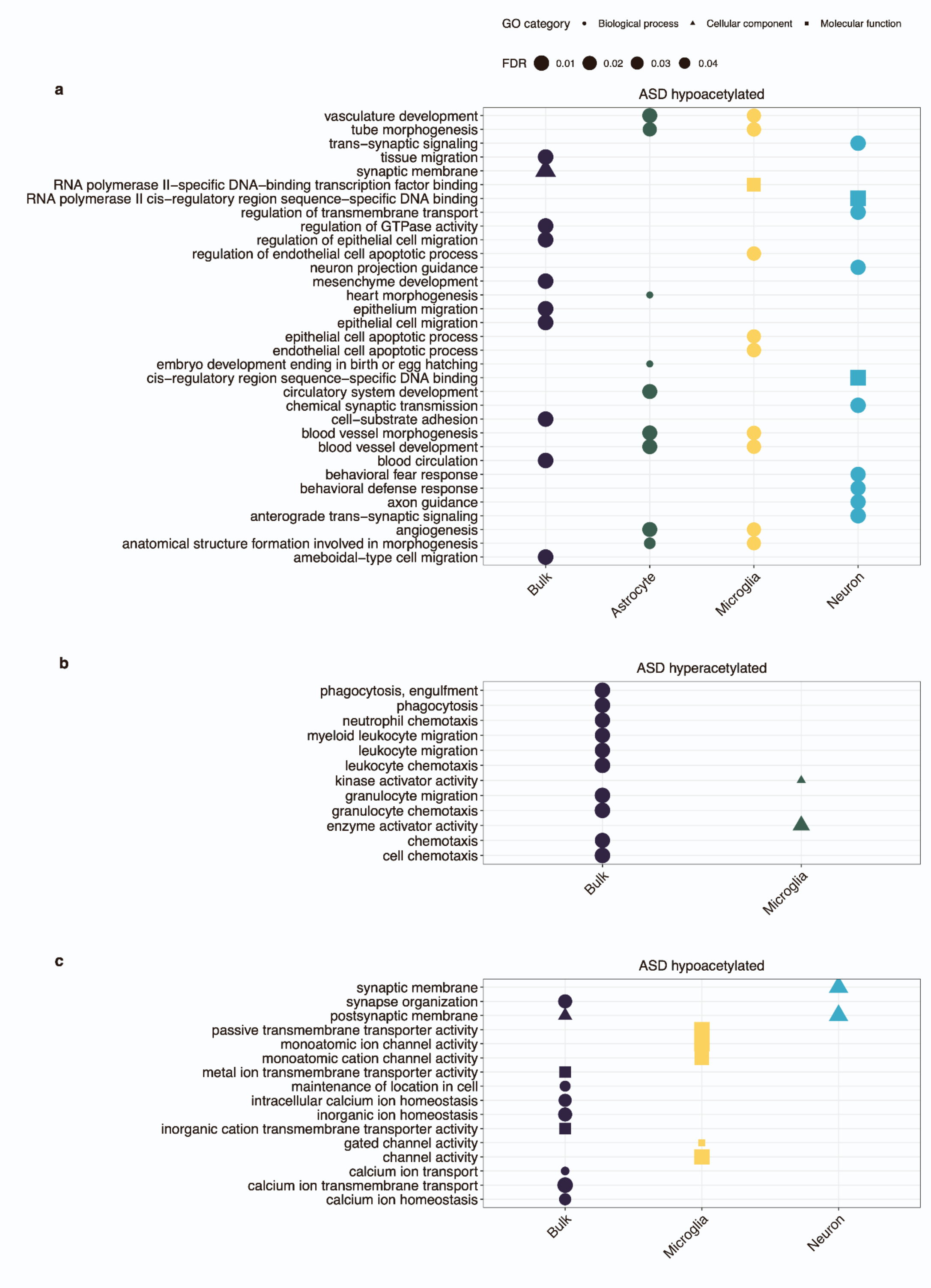

Supplementary Figure 7, related to Figure 5: Functional enrichment analysis using ASD-associated H3K27ac regions. GO enrichment analysis using ASD-associated bulk and cell type-specific **a** hypoacetylated peaks controlling for CHAS scores, **b** hyperacetylated peaks controlling for MF-derived proportions, and **c** hypoacetylated peaks controlling for MF-derived proportions. Shown are the top 10 enriched pathways for each cell type. P values were corrected using FDR.

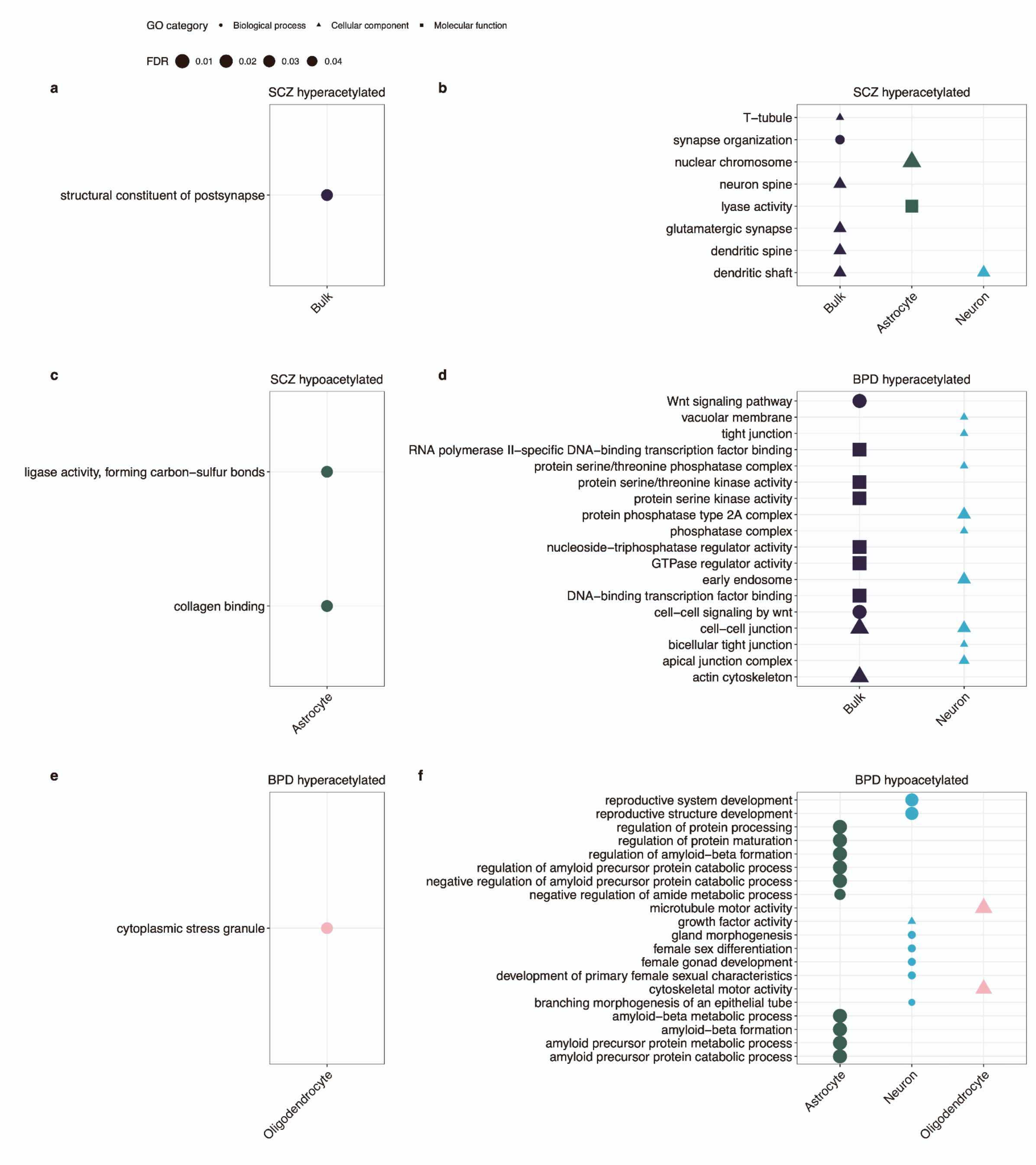

Supplementary Figure 8, related to Figure 6: Functional enrichment analysis using schizophrenia-associated and bipolar disorder-associated H3K27ac regions. a-c Pathway enrichment analysis using schizophrenia-associated bulk and cell type-specific a hyperacetylated peaks controlling for CHAS scores, (**b**) hyperacetylated peaks controlling for MF-derived proportions, and (**c**) hypoacetylated peaks controlling for MF-derived proportions. **d-f** Pathway enrichment analysis using bipolar disorder-associated bulk and cell type-specific (**d**) hyperacetylated peaks controlling for CHAS scores, (**e**) hyperacetylated peaks controlling for MF-derived proportions, and (**f**) hypoacetylated peaks controlling for MF-derived proportions. Each plot shows the top 10 enriched pathways for each cell type. P values were corrected using FDR.

References

1. Guintivano, J., Aryee, M. J. & Kaminsky, Z. A. A cell epigenotype specific model for the correction of brain cellular heterogeneity bias and its application to age, brain region and major depression. *Epigenetics* **8**, 290–302 (2013).
2. Marzi, S. J. *et al.* A histone acetylome-wide association study of Alzheimer's disease identifies disease-associated H3K27ac differences in the entorhinal cortex. *Nat. Neurosci.* **21**, 1618–1627 (2018).
3. Hunt, G. J., Freytag, S., Bahlo, M. & Gagnon-Bartsch, J. A. dtangle: accurate and robust cell type deconvolution. *Bioinformatics* **35**, 2093–2099 (2019).
4. Girdhar, K. *et al.* Chromatin domain alterations linked to 3D genome organization in a large cohort of schizophrenia and bipolar disorder brains. *Nat. Neurosci.* **25**, 474–483 (2022).
5. Kozlenkov, A. *et al.* A unique role for DNA (hydroxy)methylation in epigenetic regulation of human inhibitory neurons. *Sci. Adv.* **4**, eaau6190 (2018).
6. Yu, G., Wang, L.-G. & He, Q.-Y. ChIPseeker: an R/Bioconductor package for ChIP peak annotation, comparison and visualization. *Bioinformatics* **31**, 2382–2383 (2015).
7. Toker, L. *et al.* Genome-wide histone acetylation analysis reveals altered transcriptional regulation in the Parkinson's disease brain. *Molecular Neurodegeneration* vol. 16 Preprint at https://doi.org/10.1186/s13024-021-00450-7 (2021).
8. Sun, W. *et al.* Histone Acetylome-wide Association Study of Autism Spectrum Disorder. *Cell* **167**, 1385–1397.e11 (2016).
